# Enzymatic and molecular characterization of insecticide resistance mechanisms in field populations of *Aedes aegypti* from Selangor, Malaysia

**DOI:** 10.1186/s13071-019-3472-1

**Published:** 2019-05-16

**Authors:** Cherng-Shii Leong, Indra Vythilingam, Jonathan Wee-Kent Liew, Meng-Li Wong, Wan Sulaiman Wan-Yusoff, Yee-Ling Lau

**Affiliations:** 1 Department of Parasitology, Faculty of Medicine, University Malaya, Kuala Lumpur, 50603 Malaysia; 2 The Tropical Infectious Diseases Research and Education Centre (TIDREC), Department of Medical Microbiology, Faculty of Medicine, University of Malaya, Kuala Lumpur, Malaysia

**Keywords:** *Aedes aegypti*, *Kdr* mutation, Bioassays, Biochemical, Synergists

## Abstract

**Background:**

Dengue is a serious public health problem worldwide, including in Selangor, Malaysia. Being an important vector of dengue virus, *Aedes aegypti* are subjected to control measures which rely heavily on the usage of insecticides. Evidently, insecticide resistance in *Ae. aegypti*, which arise from several different point mutations within the voltage-gated sodium channel genes, has been documented in many countries. Thus, this robust study was conducted in all nine districts of Selangor to understand the mechanisms of resistance to various insecticides in *Ae. aegypti.* Mosquitoes were collected from dengue epidemic and non-dengue outbreak areas in Selangor.

**Methods:**

Using the Center for Disease Control and Prevention (CDC) bottle assays, the insecticide resistance status of nine different *Ae. aegypti* strains from Selangor was accessed. Synergism tests and biochemical assays were conducted to further understand the metabolic mechanisms of insecticide resistance. Polymerase chain reaction (PCR) amplification and sequencing of the IIP-IIS6 as well as IIIS4-IIIS6 regions of the sodium channel gene were performed to enable comparisons between susceptible and resistant mosquito strains. Additionally, genomic DNA was used for allele-specific PCR (AS-PCR) genotyping of the gene to detect the presence of F1534C, V1016G and S989P mutations.

**Results:**

Adult female *Ae. aegypti* from various locations were susceptible to malathion and propoxur. However, they exhibited different levels of resistance against dichlorodiphenyltrichloroethane (DDT) and pyrethroids. The results of synergism tests and biochemical assays indicated that the mixed functions of oxidases and glutathione S-transferases contributed to the DDT and pyrethroid resistance observed in the present study. Besides detecting three single *kdr* mutations, namely F1534C, V1016G and S989P, co-occurrence of homozygous V1016G/S989P (double allele) and F1534C/V1016G/S989P (triple allele) mutations were also found in *Ae. aegypti*. As per the results, the three *kdr* mutations had positive correlations with the expressions of resistance to DDT and pyrethroids.

**Conclusions:**

In view of the above outcomes, it is important to seek new tools for vector management instead of merely relying on insecticides. If the latter must be used, regular monitoring of insecticide resistance should also be carried out at all dengue epidemic areas. Since the eggs of *Ae. aegypti* can be easily transferred from one location to another, it is probable that insecticide-resistant *Ae. aegypti* can be found at non-dengue outbreak sites as well.

**Electronic supplementary material:**

The online version of this article (10.1186/s13071-019-3472-1) contains supplementary material, which is available to authorized users.

## Background

Dengue is a mosquito-borne disease which has now become a global problem owing to rapid urbanisation as well as cheapness and ease of travel [1, 2]. Currently, the incidence of dengue is about 390 million [3] in 128 countries [4]. This is a 30-fold increase in dengue cases compared to 50 years ago [5]. Malaysia is no exception as the cases of dengue have increased over the years. In 2018 (until 22nd December), 78,066 dengue cases were reported in Malaysia [6], a 77-fold increase compared to the first epidemic which occurred in 1973 [7]. In Malaysia, the state of Selangor, which is the most developed and densely-populated state, has the highest number of dengue cases (47,711 cases) [6].

The hallmark of the dengue control programmes in most countries are fogging and ultra low-volume (ULV) sprays when cases of dengue are reported [8]. It has been established that ULV is not very effective and that the insecticide droplets only get carried as far as the living rooms, whereas the mosquitoes tend to rest in the bedrooms or bathrooms [9–11]. However, due to frequent outbreaks and lack of manpower, ULV sprays must be carried out to cover larger areas.

Owing to the excessive utilization of insecticides in agriculture and public health, mosquitoes are developing resistance to the currently used insecticides [12, 13]. Most countries in Southeast Asia have reported mosquito resistance to the most commonly employed pyrethroids [14–19], but these vectors are still susceptible to organophosphates [15, 18, 20]. However, it is difficult to rely on the above results as the standard procedures have not always been followed. There is only a limited number of insecticides in our armamentarium for use in public health [21]. Pyrethroids are a common class of insecticides being used in vector control strategies and it has been shown that there is cross-resistance between pyrethroids and organochlorines [22]. Thus, insecticides should be used judiciously to prevent resistance in vectors.

Bioassays were among the first methods for the detection of resistance in mosquitoes [23]. This method employs a simple procedure, so control programmes can monitor resistance levels with ease. Subsequently, synergists were discovered to be able to improve the efficacy of the insecticides [24] by inhibiting the enzymes that were involved in detoxification of the insecticides.

It is also known that metabolic resistance owing to the detoxification of enzymes like esterases (ESTs), mixed-function oxidisases (MFO), glutathione S-transferases (GST), and acetylcholinesterases (AChE) are associated with insecticide resistance [18, 25, 26]. Generally, EST and AChE play important roles in organophosphate and carbamate resistance, GST and MFO play important role in organochlorine (DDT) and pyrethroid resistance [25]. Voltage-gated sodium channels are integral transmembrane proteins responsible for the rapidly rising phase of action potentials, and they are crucial for electrical signalling in most excitable cells [27]. Sodium channels are thus primary target of DDT and synthetic pyrethroids [27]. Due to intensive use of insecticides, *kdr* (knockdown resistance) have developed in mosquitoes [19, 28–30]. This mechanism has reduced the sodium channel sensitivity to pyrethroids and DDT, *via* one or more point mutations in the sodium channel protein [27].

Since dengue cases are increasing by the year in Selangor, Malaysia, fogging and ULV are being carried out on a regular basis. Thus, it is highly important for the overseers of control programmes to be aware of the effectiveness of these chemicals against *Ae. aegypti.* Limited studies have been carried out in Malaysia [16, 19, 31] and thus, this study was conducted in all nine districts of the state to understand the resistance mechanisms to various insecticides in *Ae. aegypti*. The mosquitoes were collected from dengue-epidemic (dengue reported every year; *Aedes* mosquito populations high during dengue outbreak season) as well as non-dengue outbreak areas. The present study, according to the authors’ knowledge, represents the first attempt to investigate the biochemical and molecular basis of insecticide resistance mechanisms in *Ae. aegypti* from dengue epidemic and non-dengue outbreak areas from Selangor. The outcome of the present study will be of importance when selecting the insecticides for application against *Ae. aegypti*, since use of chemicals are extensively practiced in vector control.

## Methods

### Study site

**Fig. 1:**
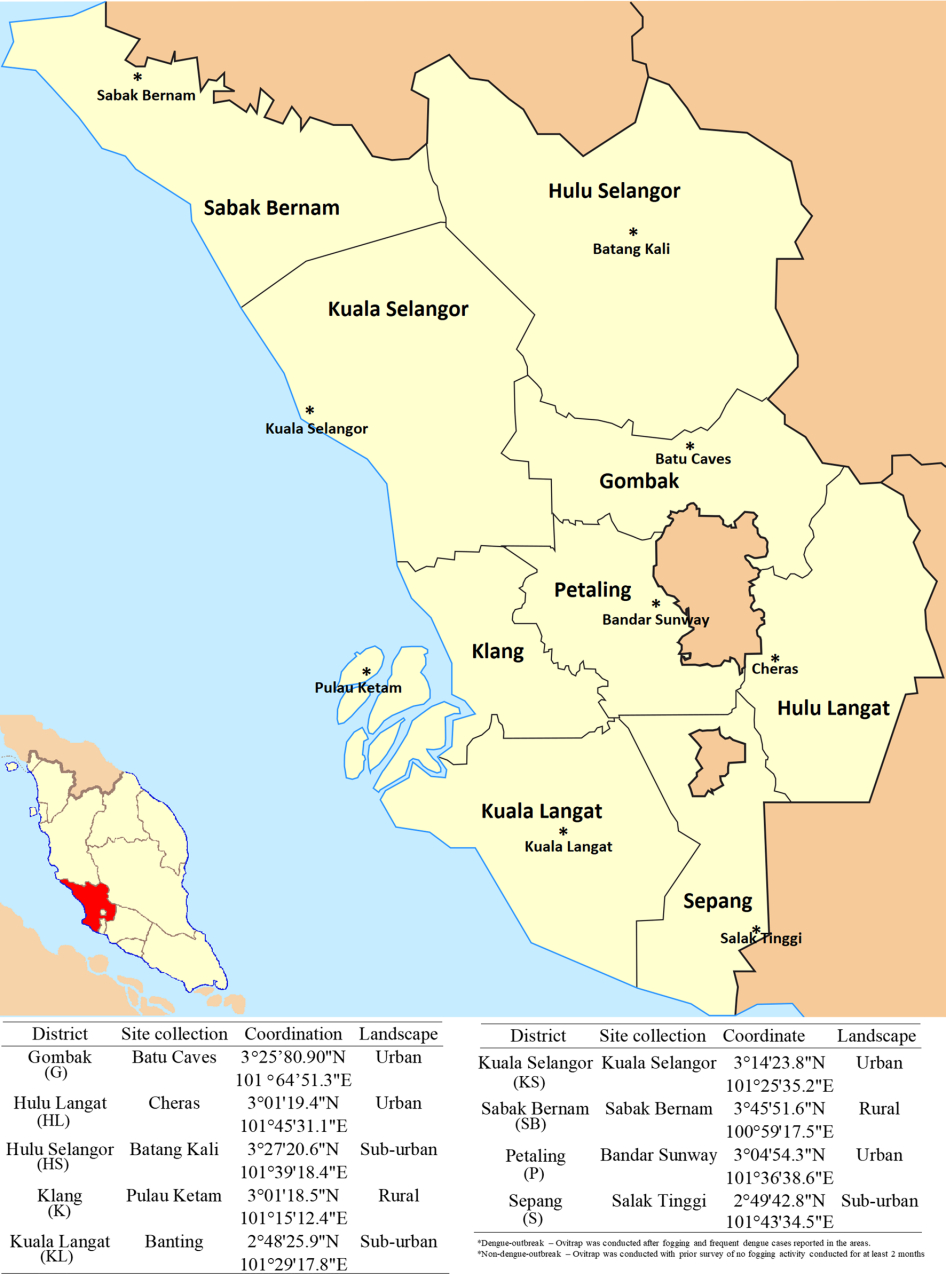
Map of Selangor showing the nine districts and collection sites of the mosquitoes

Selangor is located at the center of Peninsular Malaysia, and it serves as the main transportation hub of the country. It is also the most populated and well-developed state in Malaysia. Twenty-three percent of the total gross domestic product (GDP) of Malaysia is contributed by Selangor [32]. Collection of *Ae. aegypti* from all nine districts of the state was performed from September 2015 to April 2016 using ovitraps (Fig. 1). The nine districts are Hulu Selangor (HS), Gombak (G), Hulu Langat (HL), Kuala Langat (KL), Kuala Selangor (KS), Petaling (P), Klang (K), Sabak Bernam (SB) and Sepang (S). Forty ovitraps were set each week for three continuous weeks in each district. The traps were set at a distance of at least 20 m from each other. The traps were checked weekly. The selection of study sites was based on their dengue outbreak and non-dengue outbreak status. The eggs collected from each site were hatched in the laboratory.

All emerged adult mosquitoes were identified and segregated according to species using morphological characteristics [33]. *Aedes aegypti* colonies were maintained in standard insectary conditions (27 ± 2 °C, 75 ± 5% relative humidity, 10:14 h light:dark photocycle). Ten percent sucrose solution with vitamin B complex was provided as food to the mosquitoes. Five-to-seven-day-old adult female mosquitoes were provided with blood meals (using live white mice) for breeding purposes. Each field colony was established from about 500–1000 mosquitoes. F1 or F2 generation were used for all studies. *Aedes aegypti* of Bora-bora strain served as the reference susceptible strain. The Bora-bora strain has been maintained in the insectary for 134 generations without any exposure to insecticides.

### Tested insecticides

The present study employed all four major classes of neurotoxic insecticides, namely pyrethroids (cyfluthrin 99.8%, deltamethrin 99.6%, etofenprox 97.7%, lambda cyhalothrin 97.8% and permethrin 98.1%), organophosphates (malathion 98.7%), carbamates (propoxur 99.8%) and organochlorines (dichlorodiphenyltrichloroethane; DDT 98%). All insecticides were purchased from Sigma-Aldrich (Darmstadt, Germany).

### CDC adult bioassays

The CDC bottle bioassays were conducted as described by Brogdon and Chan [34]. To determine the diagnostic dosage and time for each insecticide, Bora-Bora strain was used as reference. The diagnostic dosage and time were used to evaluate the resistance thresholds against all field strains. Each test consisted of three insecticide-treated bottles and one ethanol-treated bottle as a control. These tests were conducted for three consecutive days (9 replicates in total). Each bottle was prepared according to Brogdon & Chan [34]. Briefly, 20–25 three-to-five-day-old sucrose-fed female *Ae. aegypti* were introduced into each 250 ml bottle coated with the diagnostic dosage of each test insecticide. The number of dead mosquitoes was recorded at one-minute intervals for a maximum of 2 h. Mosquitoes that were incapable of flying or maintaining an upright posture were considered dead. Live mosquitoes were further transferred to a paper cup with netting and 10% sucrose solution was provided. The final mortality was recorded 24 h after treatment. The diagnostic dosage was determined according to rapid end-point assays to determine the doses which killed 100% of susceptible mosquitoes within 30 min to 1 h. Table 1 shows the diagnostic dosages and resistance thresholds (time). After 24 h, dead mosquitoes, including those that were alive without the capability of coordinated movement, were labelled as susceptible (S). The survivors were labelled as resistant (R). Thirty samples of mosquitoes each susceptible and resistant to DDT and pyrethroids were randomly selected for *kdr* mutation detection using allele-specific polymerase chain reaction (AS-PCR).

**Table 1:** Diagnostic dosage and diagnostic time of CDC bottle assays based on Bora-Bora strain

Insecticide	Diagnostic dose (µg/bottle)	Diagnostic time (min)
DDT	150	45
Propoxur	1	45
Malathion	50	35
Permethrin	1	35
Etofenprox	5	35
Deltamethrin	0.5	35
Cyfluthrin	0.5	45
Lambdacyhalothrin	3	35

### Synergism tests

In order to evaluate the capability of *Ae. aegypti* to detoxify insecticides, synergism tests were performed against all field strains. The synergism tests were performed as described by Brogdon & Chan [34]. Three synergists, piperonyl butoxide 99% (PBO), S.S.S-tributyl phosphorotrithioate 97.2% (DEF), and ethacrynic acid 99% (EA) were purchased from Sigma-Aldrich for use in this study. The maximum sublethal concentration of each synergist was determined by a trial-and-error series of sublethal dosages which were administered on the reference strain. The sublethal dosage of adult synergism tests were 160 µg/bottle, 37.5 µg/bottle, and 16 µg/bottle for PBO, DEF and EA, respectively. The adult synergism tests were conducted in a manner similar to that of the CDC bottle assays, except that the female mosquitoes were exposed to the synergist-coated bottle for 1 h before being exposed to the insecticide-coated bottle; while the control was performed using ethanol-coated bottle. Each synergist was used in combination with all insecticides.

### Biochemical assays

Biochemical assays were performed to determine if the observed insecticide resistances in the Selangor *Ae. aegypti* population were due to elevated enzymatic activities. To determine the differences in the enzyme levels of individual adult female *Ae. aegypti,* biochemical assays of the susceptible strain (Bora-Bora) and field strain were performed as described by Hemingway & Brogdon [35] with minor modifications. The adult mosquitoes from the nine different districts in Selangor were individually assayed for α-EST, β-EST, AChE, GST and MFO enzymatic activities. Briefly, three-to-five-day-old female mosquitoes were individually homogenized in 200 µl of distilled water (on ice). Then, 25 µl of homogenate was pipetted for AChE assay. The remaining homogenate was centrifuged at 14,000× *rpm* at 4 °C for one minute, and the supernatant used as an enzyme source for all other enzyme assays. In total, 94 female mosquitoes from each site were assayed. All assays were conducted in duplicates using 96-well microplates. The absorbances [optical density (OD) values] were measured using the Infinite M200Pro microtitre plate reader (Tecan Trading AG, Männedorf, Switzerland). The assay for each enzyme and the enzymatic activities were calculated as described below.

### AChE assay

Some 145 µl of Triton phosphate buffer and 10 µl of 0.01 M dithiobis 2-nitrobenzoic acid solution were added to 25 µl of mosquito homogenate. This was followed by the addition of 25 µl of 0.01 M acetylthiocholine iodide to initiate the reaction. One reaction was inhibited via the addition of 0.05 µl of 0.1 M propoxur while the other was allowed to progress. After 1 h of incubation at room temperature, the reactions were measured at 405 nm absorbance. The AChE activity was calculated with respect to the percentage of insensitivity to AChE activity after propoxur inhibition [36].

### Non-specific esterase assay

Twenty µl of supernatant from the mosquito homogenates was added in duplicates to each well. To one set of samples, 200 µl of 30 mM α-naphthyl acetate was added, while to the other, 200 µl of 30 mM β-naphthyl acetate was added. The plate was incubated for 15 min at room temperature. After incubation, 50 µl of fast-blue stain was added to each well. The mixture was allowed to incubate for another 15 min, following which the OD values were measured at 570 nm. The EST activity against each substrate was calculated based on the standard curves of absorbance for known concentrations of α-naphthol or β-naphthol. The enzymatic activities were expressed as nmol of α-naphthol or β-naphthol/min/mg protein.

### GST assay

First, 10 µl of supernatant from the mosquito homogenates was added to a mixture of 200 µl 63 mM 1-chloro-2,4-dinitrobenzene (CDNB) and 10 mM reduced glutathione. The plate was allowed to incubate for 20 min at room temperature before the OD values were measured at 340 nm absorbance. Beer’s Law (A = _€_cl) was employed in the calculation of GST activity, which was expressed as CDNB/min/mg protein. The OD value (A) was transformed into µmol of CDNB conjugates using the extinction coefficient (_€_) of 4.39 mM^−1^. The path length (i.e. the depth of the buffer solution in the microplate well) was 0.6 cm.

### MFO assay

A total of 2 µl of supernatant was added to the duplicate wells. To initiate the assay, 80 µl of 0.625 M potassium phosphate buffer at pH 7.2 was added to each well, followed by 200 µl of 3,3,5,5-tetramethylbenzidine (TMBZ) (with methanol as the solvent) and 25 µl of 3% hydrogen peroxide. The mixture was incubated at room temperature for 2 h, after which the OD value was measured at 650 nm absorbance. The MFO activity was calculated from the standard curve of absorbance for known concentrations of cytochrome C [37]. The enzymatic activity was expressed as equivalent units of cytochrome P450/min/mg protein.

### Protein assay

Owing to size variances between individual mosquitoes, the analyses of all enzyme activities were corrected using the protein concentration as a standard correction factor. The bovine serum albumin standard curve was obtained using a commercial protein assay kit (Bio-Rad, Hercules, California, USA). Subsequently, the protein concentration was transformed and calculated based on the same. The protein assay was conducted by mixing 300 µl of Bio-Rad dye reagent with 10 µl of mosquito homogenate, after which the mixture was allowed to incubate for 5 min at room temperature. The plate was read at an OD of 570 nm.

### DNA extraction

Thirty adult *Ae. aegypti* each resistant and susceptible to DDT and pyrethroids from each site were subjected to molecular analysis. DNA was extracted from each specimen using the DNeasy® Blood & Tissue Kit (Qiagen, Düsseldorf, Germany). All isolation steps were conducted according to the instructions of the manufacturer.

### Allele-specific PCR (AS-PCR) detection of V1016G, F1534C, and S989P mutation

There are three *kdr* point mutations that confer pyrethroids resistance to *Ae. aegypti* namely, F1534C, V1016G and S989P [38]. These *kdr* mutations are widespread in Southeast Asia [19, 29, 39, 40]. In Malaysia point mutations of F1534C and V1016G but not S989P mutation have been detected [19]. Therefore, this study aims to detect S989P *kdr* point mutation in Malaysia.

In order to determine the associations of F1534C, V1016G, and S989P mutations with organochlorine and pyrethroid resistance, 30 randomly-selected mosquitoes from each susceptible and resistant field strain were subjected to AS-PCR. The F1534C AS-PCR was performed according to Yanola et al. [39]. Each reaction was performed in a volume of 10 µl with final concentrations of 1.5 mM MgCl_2_, 1× PCR buffer (Promega, Madison, Wisconsin, USA), 0.5 µM Phe forward primer (5′-GCG GGC TCT ACT TTG TGT TCT TCA TCA TAT T-3′), 0.165 µM Cys forward primer (5′-GCG GGC AGG GCG GCG GGG GCG GGG CCT CTA CTT TGT GTT CTT CAT CAT GTG-3′), 0.5 µM common reserve primer (5′-TCT GCT CGT TGA AGT TGT CGA T-3′), 200 µM dNTP mixture (Promega), 1 U Taq polymerase (Promega), and 25–100 ng of genomic DNA. The PCR reaction was performed at 95 °C for 2 min (initial denaturation), followed by 35 cycles of the following: 95 °C for 30 s (denaturation), 60 °C for 30 s (annealing) and 72 °C for 30 s (extension). Subsequently, final extension was performed at 72 °C for 2 min. The amplified PCR products were loaded onto a 3% agarose gel pre-stained with SYBR Safe^TM^ DNA stain (Invitrogen, Carlsbad, California, USA). Gel electrophoresis was run at 100 V for 45 min in 0.5× TBE buffer.

The V1016G AS-PCR was performed as per Stenhouse et al. [40]. Each reaction was performed in a final volume of 10 µl with final concentrations of 1.5 mM MgCl_2_, 1× PCR buffer (Promega), 0.25 µM forward primer (5′-ACC GAC AAA TTG TTT CCC-3′), 0.125 µM of each reverse primer specific for either Gly (5′-GCG GGC AGG GCG GCG GGG GCG GGG CCA GCA AGG CTA AGA AAA GGT TAA CTC-3′) or Val (5′-GCG GGC AGC AAG GCT AAG AAA AGG TTA ATT A-3′), 200 µM dNTP mixture (Promega), 1 U Taq polymerase (Promega), and 25–100 ng of genomic DNA.

PCR was carried out on a Bio-rad MyCycler^TM^ Thermal Cycle (Hercules, California, USA). The PCR conditions included an initial denaturation of 94 °C for 2 min, followed by 35 cycles of the following: 94 °C for 30 s (denaturation), 55 °C for 30 s (annealing) and 72 °C for 30 s (extension). Subsequently, final extension was performed at 72 °C for 2 min. Since the primers used in this study had GC-rich tails of varying lengths, the amplified products could be differentiated by size (i.e. 60 bp for Val and 80 bp for Gly). The amplified PCR products were loaded onto a 5% agarose gel pre-stained with SYBR Safe^TM^ DNA stain (Invitrogen). Gel electrophoresis was run at 100 V for 50 min in 0.5× TBE buffer.

A modified S989P AS-PCR was performed in accordance with the protocol of Li et al. [41]. Each reaction was performed in a final volume of 10 µl with final concentrations of 1.5 mM MgCl_2_, 1× PCR buffer (Promega), 0.4 µM M1-F common forward primer (5′-AAT GAT ATT AAC AAA ATT GCG C-3′), 0.2 µM M1-S specific forward primer (5′-GCG GCG AGT GGA TCG AAT-3′) or 0.2 µM M1-P specific forward primer (5′-GCG GCG AGT GGA TCG AAC-3′), with 0.6 µM M2-Rev common reverse primer (5′-GCA CGC CTC TAA TAT TGA TGC-3′), 200 µM dNTP mixture (Promega), 1 U Taq polymerase (Promega), and 25–100 ng of genomic DNA. The PCR reaction was performed at 94 °C for 3 min (initial denaturation) and followed by 35 cycles of the following: 94 °C for 30 s (denaturation), 60 °C for 30 s (annealing) and 72 °C for 1 min (extension). Subsequently, final extension was performed at 72 °C for 7 min. The amplified PCR products were loaded onto a 1.5% agarose gel pre-stained with SYBR Safe^TM^ DNA stain (Invitrogen). Gel electrophoresis was run at 100 V for 45 min in 0.5× TBE buffer.

### Amplification and DNA sequencing of a fragment of *Ae. aegypti* voltage-gated sodium channel gene

To confirm the AS-PCR results, amplification of DNA was conducted as per Yanola et al. [39]. The primer IIP_F (5′-GGT GGA ACT TCA CCG ACT TC-3′) was used with IIS_R (5′-GGA CGC AAT CTG GCT TGT TA-3′) to encompass the region with V1016G and S989P mutations in the IIP-IIS6 region within exons 16 to 17. The amplified product size was 581 bp. On the other hand, the F1534C mutation was sequenced using the primers GE-IIIS6_F (5′-GCT GTC GCA CGA GAT CAT T-3′) with IIIS6_R (5′-GTT GAA CCC GAT GAA CAA CA-3′) which amplified the IIIS4-IIIS6 region within the exons 24–26. The amplified product’s size was 635 bp.

PCR was carried out in a reaction volume of 50 µl, which contained 1.5 mM MgCl_2_, 1× PCR buffer (Promega), 0.5 µM forward and reverse primers, 200 µM dNTP mixture (Promega), 1 U Taq polymerase (Promega), and 25–100 ng of genomic DNA. The amplification consisted of an initial heat-activation step of 95 °C for 2 min, followed by 35 cycles of the following: 95 °C for 30 s, 63 °C for 30 s and 72 °C for 30 s. Final extension was done at 72 °C for 2 min.

The amplified PCR products were loaded onto a 1.5% agarose gel pre-stained with SYBR Safe^TM^ DNA stain (Invitrogen, USA), after which gel electrophoresis was run at 100 V for 45 min in TBE buffer. The gel was viewed under UV-light, after which the designated band was cut out, placed inside a 1.5 ml microcentrifuge tube, and stored in − 20 °C until required for sequencing. DNA sequencing of the PCR products was performed using the service provided by Genomics BioScience and Technology Co. Ltd. (New Taipei City, Taiwan), which employed a BigDye® Terminator v3.1 in ABI PRISM® 3730*xl* DNA Analyzer (Applied Biosystems, Foster City, California, USA). Forward and reverse sequencing reactions were done using the forward and reverse PCR primers as mentioned above. All sequence analyses and editing were performed using the BioEdit Sequence Alignment Editor v7.2.3. Both forward and reverse nucleotide sequences were aligned, and a consensus sequence was formed for each sample. Only sequences of good quality were trimmed for further analysis. The trimmed sequences were then aligned using Clustal W, along with other similar sequences available in GenBank. All sequences generated in the present study were deposited in the GenBank database under the accession numbers MK005552-MK005584. For the 1016 and 989 mutation-point analyses, the included sequences were: MF794972 (V1016V, F1534F homozygous allele); MF794974 (V1016V, F1534C homozygous allele); MF794978 (V1016V/G heterozygous, F1534F homozygous allele), MF794984 (G1016G, F1534F homozygous allele) [42]; KY057038 (V1016G homozygous allele), KY057037 (S989P homozygous allele) [28]; and AB914689 (V1016G, S989P homozygous allele) [43]. As for the F1534C mutation analysis, sequences AB914688 (F1534F homozygous allele), AB914687 (F1534C homozygous allele) [43], EU259810 (DDT-resistant, F1534F homozygous allele), EU259811 (DDT- and permethrin-resistant, F1534C homozygous allele) [39], and MF794990 (F1534F/C heterozygous allele) [42] were included.

### Statistical analysis

The mortality rate (%) was used to describe the susceptibility statuses of *Ae. aegypti* and was used to evaluate the effectiveness of the synergists against the toxicities of the insecticides. Mortality rates as derived from the CDC bottle bioassays were used to determine the susceptibility statuses of the field strains of *Ae. aegypti* vis-à-vis the diagnostic dosages and times of the reference strain (Bora-Bora). A mortality rate of 98–100 indicates susceptibility; 90–97 indicates tolerance/ intermediate resistance; and < 90 indicates resistance [23, 34]. The data of the CDC bottle assays which were within 5–95% were subjected to probit analyses of Finney [44] to obtain the knockdown rates, KT_50_ and KT_99_, for each insecticide. The said data were then pooled for analysis. Resistance ratios (RR_50_) were calculated by dividing the KT_50_ values for the field strain with those for the reference strain, based on the CDC bottle bioassays. The RR_50_ was used to determine the correlation between the different insecticides [45]. If the control’s mortality was between 5–20%, the percentage mortalities would be corrected by Abbott’s formula [46].

Levene’s and Kolmogorov-Smirnov tests were performed to check the normality of the knockdown rates, mortality rates and enzymatic activities. To stabilize the variances between the data, an arcsine log function was performed on data that were not normally distributed. The differences between the Bora-Bora and field strains were determined using the Mann-Whitney non-parametric test or two-sample t-test. Cross-resistances between insecticides were determined using the Spearmanʼs rank-order correlation. Independent chi-squared test was carried out to compare the differences in *Ae. aegypti* possessing V1016G, F1534C, and V1016G and S989P mutations. Statistical significance was set at *P* < 0.05. The Statistical Package for Social Sciences (SPSS; IBM SPSS Statistics 19) software was used for data analyses and interpretations, while Microsoft Excel version 2016 (Microsoft Inc.) for generating graphs.

## Results

### Bioassays

**Fig. 2:**
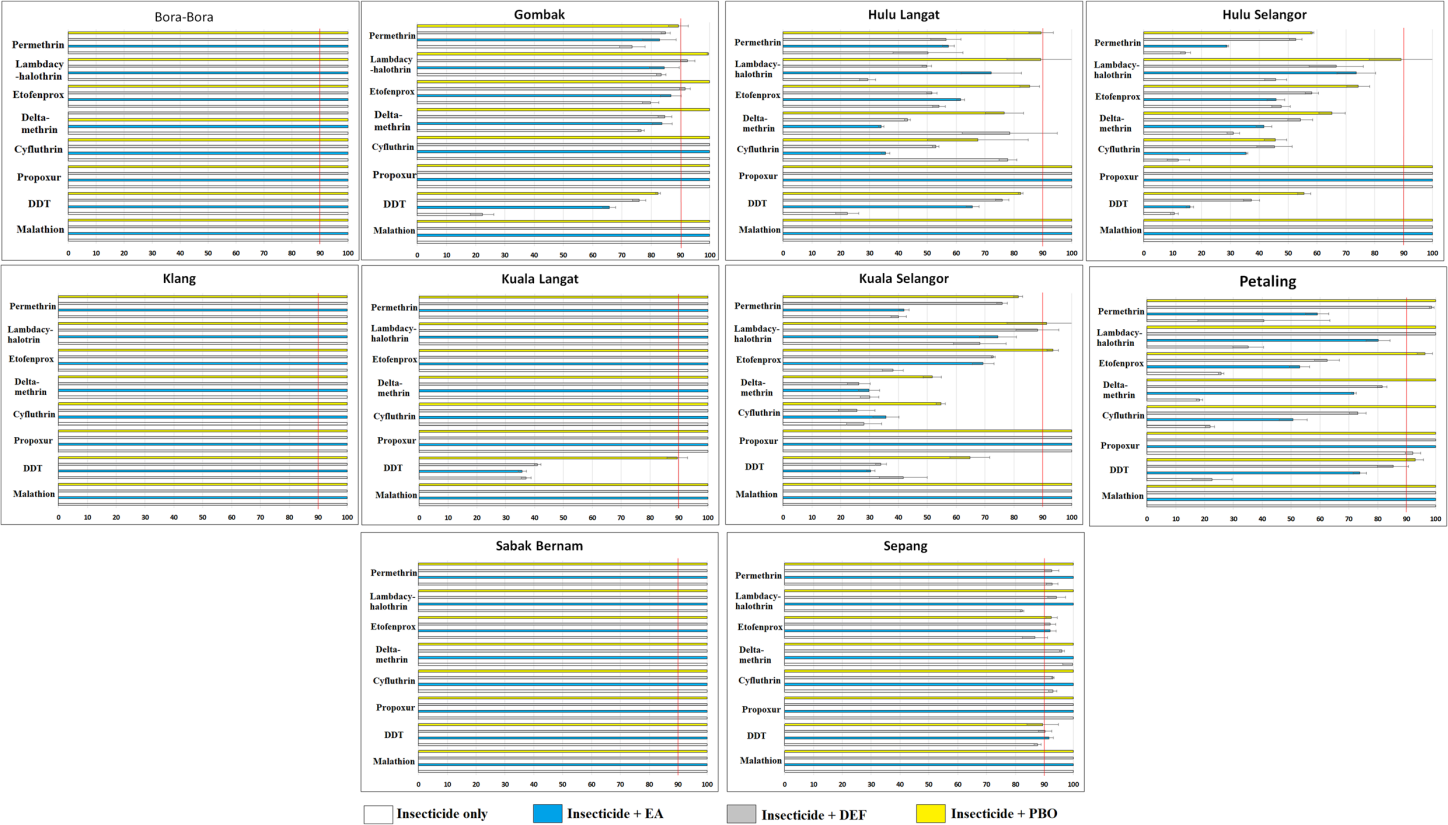
Mortality rates of adult female *Aedes aegypti* against various insecticides and synergists. Redline indicates the resistance threshold

The diagnostic dosages and diagnostic times of the different insecticides (based on Bora-Bora strain) are shown in Table 1. Female *Ae. aegypti* from all sites showed 100% mortality to malathion and propoxur within 2 h of exposure, except for the Petaling strain which recorded 92.22% mortality 24 h post-treatment with propoxur (Fig. 2) (Additional file 1: Table S1). The Klang strain was susceptible to all test insecticides, with 100% mortality achieved within the diagnostic time. As for DDT, all field strains exhibited resistance to it with mortality rates of < 90%, except for Sabak Bernam and Klang (100%).

**Fig. 3:**
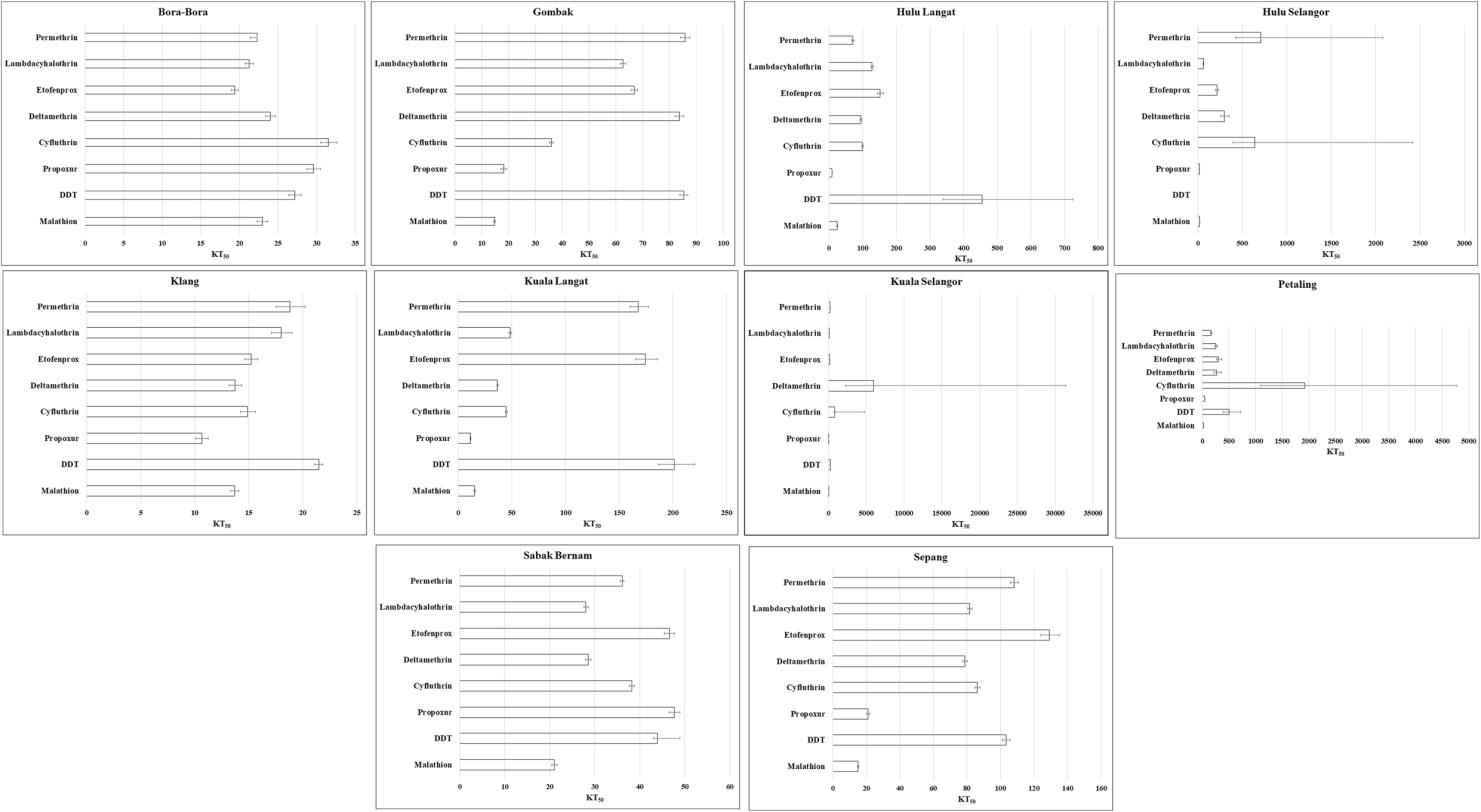
Knockdown time KT_50_ of adult female *Aedes aegypti* to various insecticides

**Fig. 4:**
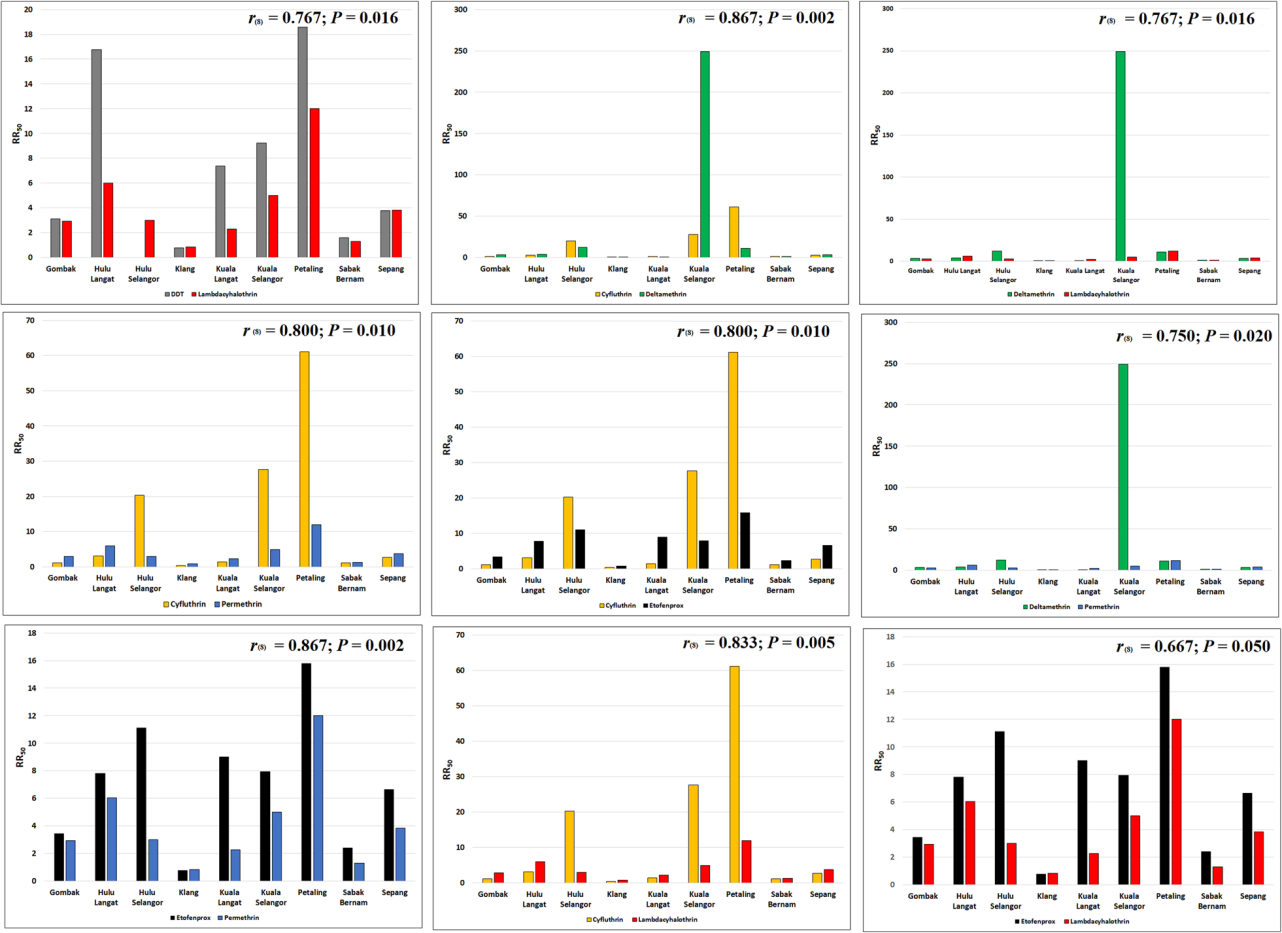
Correlation between resistance ratios of DDT and lambda cyhalothrin (**a**), cyfluthrin and deltamethrin (**b**), deltamethrin and lambda cyhalothrin (**c**), cyfluthrin and permethrin (**d**), cyfluthrin and etofenprox (**e**), deltamethrin and permethrin (**f**), etofenprox and permethrin (**g**), cyfluthrin and lambda cyhalothrin (**h**), and etofenprox and lambda cyhalothrin (**i**)

Most of the female *Ae. aegypti* field strains were resistant to all five pyrethroid insecticides, except for Klang and Sabak Bernam strains which recorded 100% mortality 24 h post-treatment. However, the Gombak strain had 100% mortality to cyfluthrin while the Kuala Langat strain showed 100% mortality to cyfluthrin and deltamethrin. All other strains (Hulu Langat, Hulu Selangor, Kuala Selangor and Petaling) exhibited different degrees of resistance to pyrethroids as shown in Fig. 2. The KT_50_ (Fig. 3) and KT_99_ also varied among the different field strains (Additional file 1: Table S2). Furthermore, the Spearmanʼs rank-order correlation test indicated a significant correlation between the resistance ratios of DDT and lambdacyhalothrin (*r*_(8)_ = 0.767, *P* = 0.016), cyfluthrin and permethrin (*r*_(8)_ = 0.800, *P* = 0.010), cyfluthrin and lambdacyhalothrin (*r*_(8)_ = 0.833, *P* = 0.005), cyfluthrin and deltamethrin (*r*_(8)_ = 0.867, *P* = 0.002), cyfluthrin and etofenprox (*r*_(8)_ = 0.800, *P* = 0.010), deltamethrin and permethrin (*r*_(8)_ = 0.750, *P* = 0.020), deltamethrin and lambdacyhalothrin (*r*_(8)_ = 0.767, *P* = 0.016), etofenprox and permethrin (r_(8)_ = 0.867; *P* = 0.002), as well as etofenprox and lambdacyhalothrin (*r*_(8)_ = 0.667, *P* = 0.050). There were no significant correlations between other insecticides (Fig. 4).

### Effectiveness of synergists

Synergists (DEF, EA and PBO) were investigated for their efficiency in improving the effectiveness of insecticides against mosquitoes. The mortality rates of the field strains of *Ae. aegypti* which were treated with different combinations of insecticides and synergists are shown in Fig. 2. In summary, synergists improved the efficiencies of the insecticides, but only Sabak Bernam and Sepang female *Ae. aegypti* strains exhibited 100% mortality following treatment with a combination of insecticides and synergists. The results showed that synergists increased the mortality rates of all strains of female *Ae. aegypti* against all the tested insecticides. However, most of the field female *Ae. aegypti* strains still showed resistance (i.e. < 90% mortality rate) against DDT and pyrethroids even when these have been used in combination with synergists.

### Biochemical assays

**Fig. 5:**
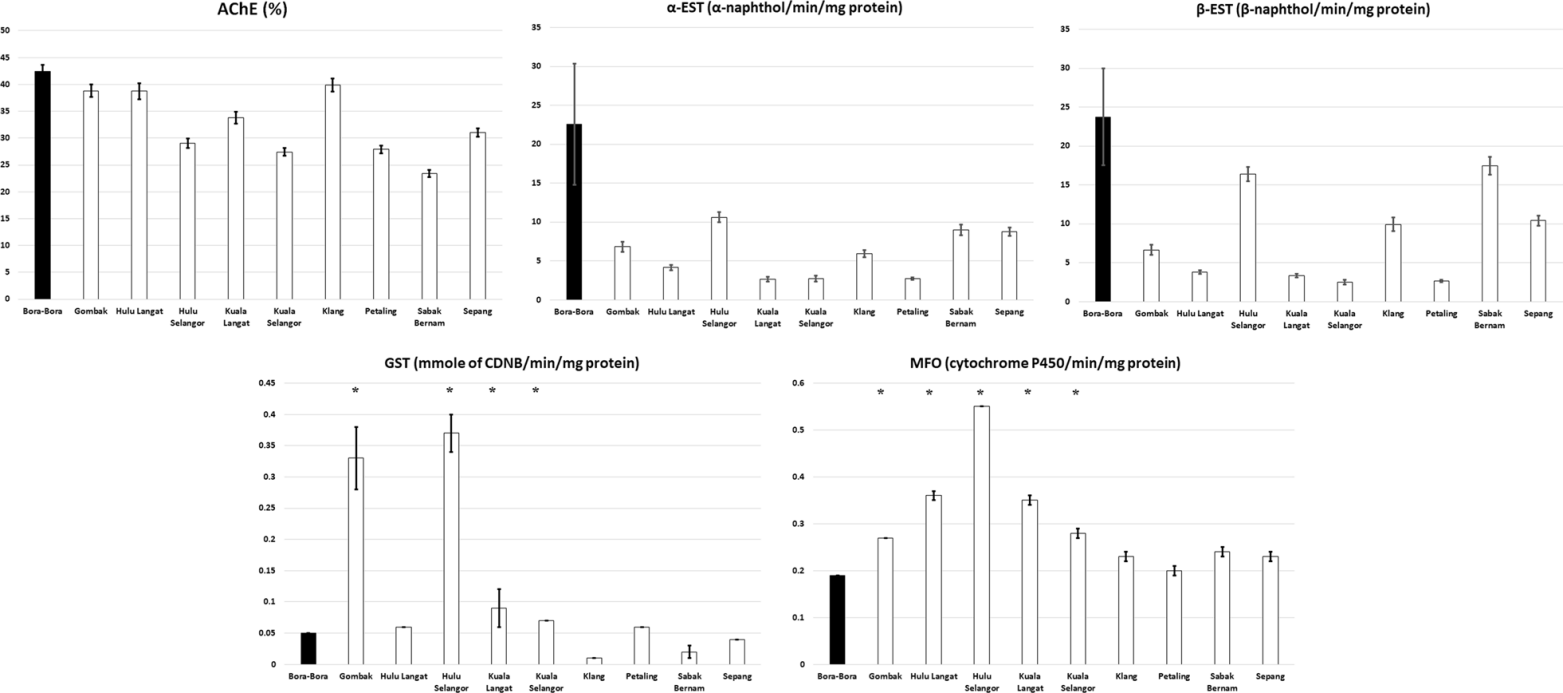
Mean (± SE) levels of insensitive acetylcholinesterase (AChE), glutathione-S-transferase (GST), non-specific esterase (α- and β-EST) and mono-oxygenase (MFO) activities of *Ae. aegypti* in Selangor. Asterisks indicate significantly higher values when compared with Bora-Bora Strain (*P* < 0.05, Mann-Whitney test)

All data were pooled and analyzed. Four strains of *Ae. aegypti* (Kuala Selangor, Kuala Langat, Hulu Selangor and Gombak) exhibited elevated levels of GST activity using Mann-Whitney U-test (Gombak *U*_(273)_ = 579.00, *Z* = − 12.40, *P* < 0.0001; Hulu Selangor *U*_(266)_ = 5677.50, *Z* = − 3.57, *P* < 0.0001; Kuala Langat *U*_(268)_ = 2126.50, *Z* = − 9.67, *P* < 0.0001; Kuala Selangor *U*_(272)_ = 1017.50, *Z* = − 11.61, *P* < 0.0001) when compared with the Bora-Bora strain as shown in Fig. 5. These four strains, along with the Hulu Langat strain, also exhibited a significant increase in MFO activity (Gombak *U*_(372)_ = 3954.50, *Z* = − 12.94, *P* < 0.0001; Hulu Langat *U*_(372)_ = 798.00, *Z* = − 15.95, *P* < 0.0001; Hulu Selangor *U*_(372)_ = 472.00, *Z* = − 16.27, *P* < 0.0001; Kuala Langat *U*_(369)_ = 268.50, *Z* = − 16.46, *P* < 0.0001; Kuala Selangor *U*_(372)_ = 12548.00, *Z* = − 4.66, *P* < 0.0001). Although significantly increased activities of GST and MFO have been observed in some field strains of *Ae. aegypti*, the Spearmanʼs rank-order correlation test did not reveal any significant correlation with other insecticides or enzymes.

### *Kdr* screening

**Fig. 6:**
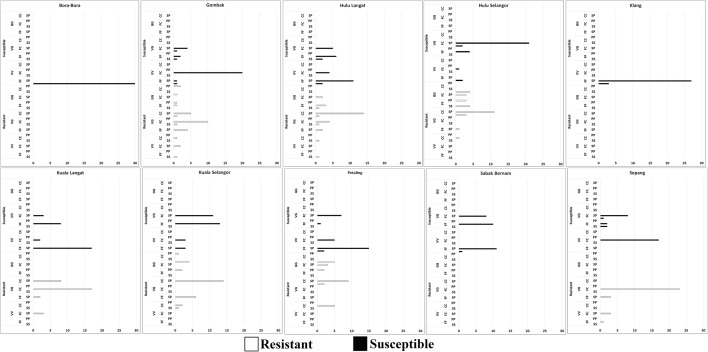
V1016G, F1534C, and S989P point mutations in *Aedes aegypti* collected from Selangor. *Abbreviations*: VV, homozygous wild type of V1016G; VG, heterozygous of V1016G; GG, homozygous mutant of V1016G; FF, homozygous wild type of F1534C; FC, heterozygous of F1534C; CC, homozygous mutant of F1534C; SS, homozygous wild type of S989P; SP, heterozygous of S989P; PP, homozygous mutant of S989P

In the present study, a cheap, reliable, and rapid AS-PCR was used to detect the *kdr* mutation as it provided results after gel electrophoresis (Additional file 2: Figure S1). The genotype and allele frequencies (Fig. 6, Additional file 1: Tables S3–S5) were derived from 270 susceptible and 210 resistant randomly-selected *Ae. aegypti* with 30 Bora-Bora strain of *Ae. aegypti*. The results show that the frequency of *Ae. aegypti* possessing the homozygous F1534C mutation with heterozygous V1016G was significantly higher than the *Ae. aegypti* possessing the homozygous V1016G with heterozygous F1534C (*χ*^2^ = 113, *df* = 2, *P* < 0.001). On the other hand, frequency of single homozygous mutation of F1534C (*χ*^2^ = 116, *df* = 2, *P* < 0.001) and V1016G (*χ*^2^ = 100, *df* = 2, *P* < 0.001) were significantly higher compared to co-occurrence of homozygous V1016G and S989P.

All resistant and susceptible mosquitoes had the three mutations genotyped for DDT and pyrethroids resistance/susceptibility (Table 2). The 1534C-mutated allele was significantly associated with DDT (*r*_(8)_ = 0.711, *P* = 0.032), cyfluthrin (*r*_(8)_ = 0.812, *P* = 0.008), deltamethrin (*r*_(8)_ = 0.845, *P* = 0.004), etofenprox (*r*_(8)_ = 0.742, *P* = 0.021) and lambdacyhalothrin (*r*_(8)_ = 0.879, *P* = 0.002) resistance. The 1016G-mutated allele significantly correlated with cyfluthrin (*r*_(8)_ = 0.783, *P* = 0.013), deltamethrin (*r*_(8)_ = 0.833, *P* = 0.005), etofenprox (*r*_(8)_ = 0.850, *P* = 0.004), lambdacyhalothrin (*r*_(8)_ = 0.817, *P* = 0.007) and permethrin (*r*_(8)_ = 0.717, *P* = 0.030) resistance. On the other hand, only permethrin resistance (*r*_(8)_ = 0.700, *P* = 0.036) was associated with the S989P-mutated allele.

**Table 2:** Correlation between resistance ratio of each insecticides and *kdr* mutations of *Ae. aegypti*

Chemical	F1534C	V1016G	S989P
DDT	*r*_(8)_ = 0.711 *P* = 0.032^*^	*r*_(8)_ = 0.483 *P* = 0.187	*r*_(8)_ = 0.300 *P* = 0.433
Cyfluthrin	*r*_(8)_ = 0.812 *P* = 0.008^*^	*r*_(8)_ = 0.783 *P* = 0.013^*^	*r*_(8)_ = 0.383 *P* = 0.308
Deltamethrin	*r*_(8)_ = 0.845 *P* = 0.004^*^	*r*_(8)_ = 0.833 *P* = 0.005^*^	*r*_(8)_ = 0.283 *P* = 0.460
Etofenprox	*r*_(8)_ = 0.745 *P* = 0.021^*^	*r*_(8)_ = 0.850 *P* = 0.004^*^	*r*_(8)_ = 0.600 *P* = 0.088
Lambdacyhalothrin	*r*_(8)_ = 0.879 *P* = 0.002^*^	*r*_(8)_ = 0.817 *P* = 0.007^*^	*r*_(8)_ = 0.167 *P* = 0.668
Permethrin	*r*_(8)_ = 0.644 *P* = 0.061	*r*_(8)_ = 0.717 *P* = 0.030^*^	*r*_(8)_ = 0.700 *P* = 0.036^*^

*Note*: Asterisk indicates significant correlation between resistance ratio of insecticides (*P* < 0.05; Spearmanʼs rank-order correlation)

The results of the present study showed that triple and double homozygous mutations were detected in a single *Ae. aegypti*. Three samples had triple homozygous mutations (Gombak-02, Gombak-11, and Kuala Selangor-08) (Additional file 2: Figures S2–S4). Double homozygous mutations of V1016G and S989P were observed in the three strains: Hulu Selangor (Hulu Selangor-02 and Hulu Selangor-14); Petaling (Petaling-02 and Petaling-24); and Kuala Selangor (Kuala Selangor-07) (Additional file 2: Figures S2, S3). In order to further confirm the presence of triple and double homozygous mutations, the IIP-IIS6 and/or IIIS4-IIIS6 regions of all samples that exhibited these mutations were sequenced.

After examining the DNA sequence chromatograms, 16 of the 20 sample nucleotide sequences for the 1016 and 989 mutation point analyses (IIP-IIS6 region), and 17 out of 20 sequences for the F1534C mutation analysis (IIIS4-IIIS6 region) exhibited clear, singular peaks, indicating good quality sequencing and no contamination. No mutations were observed in the Bora-Bora-01, Bora-Bora-02, Klang-05, Klang-09 and Klang-18 sequences, thereby supporting the AS-PCR results. Sequencing further confirmed the results of AS-PCR, whereby Kuala Selangor-01, Kuala Selangor-15, and Gombak-22 were shown to have the F1534C mutation but not S989P and V1016G. Gombak-02, Gombak-11, Hulu Selangor-02, Hulu Selangor-14, Petaling-05, Kuala Selangor-07, Kuala Selangor-08 and Petaling-24 were all homozygous for the S989P and V1016G mutations (Additional file 2: Figures S2–S4). On the other hand, Hulu Selangor-01, Hulu Selangor-02, Hulu Selangor-14 and Petaling-02 exhibited heterozygous F1534F/C mutations, while Gombak-02, Gombak-22, Gombak-11, Kuala Selangor-01, Kuala Selangor-08, Kuala Selangor-14 and Kuala Selangor-15 exhibited homozygous F1534C mutation (Additional file 2: Figure S4).

## Discussion

*Aedes aegypti* from Selangor showed various levels of resistance against organochlorine and pyrethroids. However, they exhibited susceptibility against malathion (organophosphates) and propoxur (carbamate). Organophosphates (especially malathion) have been the insecticide of choice during dengue epidemics, whereby control measures relied heavily on both thermal fogging and ULV to rapidly kill the infected *Aedes* mosquitoes [47]. However, all field *Ae. aegypti* were susceptible to the said chemical, with 100% mortality. In Malaysia, propoxur has never been used as an active ingredient in vector control programmes or public health activities. It is noteworthy that propoxur has been used as a household pest control product in the early 1970s, but its utilization was terminated in the 1990s [48]. Therefore, the resistance of *Ae. aegypti* against propoxur was low, presumably due to infrequent application of the same.

Among all the field strains, Klang and Sabak Bernam strains were most susceptible to all insecticides, with 24 h post-treatment mortalities of > 98%. Evidently, these areas have not been affected by dengue outbreaks in recent years. On the other hand, the remaining field strains of *Ae. aegypti* were resistant to DDT and pyrethroids. Pyrethroids are a major class of insecticides in the pest control industry and are widely used in dengue and malaria control programmes [49]. Although DDT has never been used for dengue control in Malaysia, it has been utilized from the late 1950s until the 1980s for malaria eradication [50]; its usage was stopped in 1998 [49]. Few studies have shown that the DDT-resistant phenotype was still present in *Ae. aegypti* (Malaysia) [51], *Culex quinquefasciatus* (Malaysia) [52], and *Anopheles darlingi* (Colombia) [53] even though DDT has no longer been used for decades. The reference strain of *Culex quinquefasciatus* exhibited resistance against DDT after being maintained in an insecticide-free insectary for 117 generations [52]. On the other hand, after 17 years of banning DDT application, *An. darlingi* in Colombia was still found to be resistant against DDT and also lambda-cyhalothrin [53]. Furthermore, DDT and pyrethroids share the same mode of action in which the voltage-gated sodium channels were targeted. Thus, the observed resistances may have been due to the extensive usage of pyrethroids in pest control and public health activities. Additionally, cross-resistances between DDT and pyrethroids [54] as well as within pyrethroids owing to the same target sites are well-known [55, 56] and these may be similarly observed in the present study. The results of this research showed that the emergence of insecticide resistance is likely to be associated with the frequency of dengue outbreaks owing to the excessive utilization of insecticides in control measures. Therefore, new strategies are urgently required to replace fogging and ULV during dengue outbreaks.

Biochemical assays demonstrated elevated levels of GSTs and MFO in some of the field strains of *Ae. aegypti*, but this was not the case for ESTs and AChE. These findings corroborated with the bioassay results, in which the field strains of *Ae. aegypti* were susceptible to malathion and propoxur but resistant to DDT and pyrethroids. Elevated GST levels are responsible for DDT resistance [57], and this was observed in the Gombak, Hulu Selangor, Kuala Langat and Kuala Selangor strains of *Ae. aegypti*. Since DDT and pyrethroids share the same target site (voltage-gated sodium channels), the observed elevation in GST level could have been due to resistance towards pyrethroids, as a result of the extensive usage of this class of insecticides in vector control programmes [57–59].

Nevertheless, the results have shown partial synergistic effects of DEF (the main inhibitor of esterases) in some of the field strains. This was probably attributable to its secondary GST-inhibitor ability [60, 61] since the biochemical assays have detected elevated GST activities in five of the field strains. Esterase (including AChE) activities are well known for conferring organophosphate and carbamate resistance in mosquitoes [48, 62]. However, the low frequency of ESTs and AChE activities in the present study showed that this mechanism was not involved. Furthermore, as per the bioassay results, the susceptibility statuses of all field strains to propoxur and malathion further supported this theory.

Pyrethroid resistance is often related to elevated MFOs levels [63, 64], as detected in Kuala Selangor, Kuala Langat, Hulu Selangor, Hulu Langat and Gombak strains of *Ae. aegypti*. Many studies have identified PBO as a MFOs inhibitor [57]. Similarly, this study has demonstrated that the addition of PBO to pyrethroids helped increase the mortality, thereby confirming the involvement of MFO in pyrethroid resistance.

Although the employment of synergists has significantly promoted the mortality of field *Ae. aegypti* as compared to the reference strain, many of the field strains still remained resistant (24 h post-treatment mortality < 90%). In addition, all field strains (except for Sabak Bernam and Klang) exhibited resistance to DDT and pyrethroids. This could also be due to cross-resistance between organochlorine and pyrethroids. Therefore, this further suggests the involvement of more than one mechanism giving rise to insecticide resistance. Additionally, a few studies have suggested that toxicological changes in arthropods were not directly correlated with enzymatic activities [65, 66]. Indeed, the evolution/mutation of multiple strains is not a new phenomenon and is becoming a serious issue worldwide. In Malaysia, evidence of pyrethroid resistance in *Ae. aegypti* has been reported [67, 68]. However, the mechanisms that conferred resistances toward these insecticides in the mosquitoes were poorly understood. Therefore, the present study has utilized AS-PCR to detect the involvement of target site insensitivity mechanism in DDT and pyrethroid resistance in *Ae. aegypti.*

This study to our knowledge is the first to describe the S989P mutation in Malaysian *Ae. aegypti*. The first report on F1534C and V1016G mutations in *Ae. aegypti* was in 2015 [19]. In the present study, low frequencies of F1534C (13.33%), V1016G (8.75%) and S989P (5.09%) mutations were found in the Selangor *Ae. aegypti*. Evidently, these mutations have also been documented in Thailand [39, 40], Singapore [29] and Myanmar [43]. The F1534C mutation was found to be significantly associated with DDT and pyrethroid resistance in the present study, in line with the outcomes of other researches [19, 43, 69]. However, other studies have only found F1534C mutation to be significantly associated with type I pyrethroid resistance [39, 40]. The contribution of F1534C to multiple-pyrethroid resistance was possibly due to the additive contribution of the V1016G mutation since the latter has been frequently reported to be responsible for pyrethroid resistance [22, 42, 70, 71] (especially type II pyrethroids) [38, 40]. Indeed, most of the pyrethroid-resistant mosquitoes with F1534C mutation also carry the V1016G mutation. It should be noted that mosquitoes with the V1016G mutation are thought to be protected from deltamethrin [40]. Statistical analyses have shown that S989P mutation is correlated with permethrin resistance only. It cannot be definitively concluded if this is so, as the effect of only S989P mutation on permethrin resistance was not directly investigated in this study. The S989P mutation can be often found co-occurring with the V1016G mutation and also F1534C mutation in the present study. Hirata et al. [70] demonstrated that S989P mutation does not affect permethrin sensitivity whereas other studies [38, 40] have been unable to provide direct evidence to justify the effect of the single S989P mutation in pyrethroid resistance. Therefore, the role of the S989P mutation in permethrin resistance needs additional confirmation as suggested by some researchers [72, 73].

This study found two co-occurrent point mutations, namely S989P/V1016G and F1534C/S989P/V1016G. However, no F1534C/V1016G mutation only was found, in congruence with the study by Ishak et al. [19] in Penang (Malaysia). The S989P mutation has been frequently linked to the V1016G mutation but sometimes, the V1016G mutation has been found in the absence of the S989P mutation [38, 40]. Both studies have reported that the co-occurrence of S989P/V1016G enhances the resistance towards deltamethrin. Similarly, Hirata et al. [70] have found that the combination of V1016G/S989P moderately reduced the sodium channel’s sensitivity to deltamethrin. Furthermore, these authors have also detected a gradual decline in the sensitivities to permethrin and deltamethrin when there was a co-occurrent F1534C/S989P/V1016G triple mutation [70] which points to the synergistic effect of the combination of mutant alleles. In addition, Plernsub et al. [74] have also reported that combinations of single *kdr* mutations led to a relatively higher level of resistance against pyrethroids. Interestingly, it has been found that the triple heterozygote (F1534C, V1016G and S989P) was resistant against deltamethrin and permethrin though exhibiting intermediate resistance compared to F1534C homozygote which has 2-fold lower resistance and S989P + G1016 homozygote which has 2-fold higher resistance. However, addition of PBO reduced their resistance by 2-fold, suggesting the partial role of oxidase enzymes in resistance [74]. In the present study, this triple heterozygous mutation was found distributed in the susceptible and resistant individuals, therefore, we agree that the oxidase may be contributing to pyrethroid resistance in the resistant triple heterozygotes. One of the limitations of this study was that genetic linkages of resistant trait among all nine study areas could not be established as sequencing was not performed on all the samples from all the nine study areas. Furthermore, there could be other point mutations on the *kdr* gene such as the G923V and D1794Y mutations, outside of the sequencing regions being studied here, that could have contributed to the variability observed [39].

In Southeast Asia, these three mutations have been reported in *Ae. aegypti* populations. However, this is the first report on triple homozygous mutations in Malaysia, even though only three samples (0.63%) were found to have the same. Similarly, studies in Myanmar [43] and Malaysia [19] have also detected a low occurrence of multiple homozygous mutations. Both studies have reported a higher resistance to pyrethroids when combinations of single *kdr* mutations were present. Moreover, an outdoor thermal fogging study, which employed a combination of deltamethrin, S-bioallethrin and PBO, has found that S989P/V1016G homozygous *Ae. aegypti* survived the spray. On the contrary, most of the F1534C homozygous *Ae. aegypti* were killed [71]. The efficiency of thermal fogging spray was most likely to be even less effective in natural situations. Hence, the present study highlights the significant impact of multiple homozygous mutations of *Ae. aegypti* on vector control programmes which utilize pyrethroid-based approaches. Notably, when this triple homozygous mutation occurs naturally in highly-resistant *Ae. aegypti*, it is timely to consider other methods for control. However, the current low occurrence of this triple homozygous mutation was most probably attributable to its low fitness as proposed by Stenhouse et al. [40] and Hirata et al. [70]. Yet, the possibility of compensatory mutations that restore fitness might enable this genotype to become more widespread, which will lead to the ineffectiveness of pyrethroids against this dengue vector. Owing to emergence of insecticide resistance in many dengue-prone countries, new strategies should be considered to prevent outbreaks [75]. It has been shown that asymptomatic persons are more infectious to *Aedes* mosquitoes [76], so the current control measures (which are instituted only after dengue cases have been reported), might perhaps be too late. Thus, early detection of dengue outbreaks, in addition to the prudent management and use of insecticides, is required to avoid an increase in dengue cases.

## Conclusions

Generally, *Ae. aegypti* from dengue outbreak areas had higher resistance to insecticides than those from non-dengue outbreak areas. The results show that organophosphates and carbamates are still suitable for use in vector control programmes. When pyrethroids are the major class of insecticides in vector control programmes, the *kdr* mutations in Malaysian *Ae. aegypti* populations contributed significantly to pyrethroid resistance, while MFO and GST enzymes had a partial role. Therefore, the development of new insecticides with novel modes of action is required to replace the conventional ones. To ensure the success of vector control, new tools for countering resistance are required. Also, innovative strategies should be constructed to inhibit the spread and evolution of resistance. It should be noted that *Ae. aegypti* eggs can easily be spread from one location to another and thus, it is postulated that the occurrence of insecticide-resistant *Ae. aegypti* might also occur in non-dengue outbreak areas in the future.

## Additional files

**Additional file 1: Table S1.** Knockdown rate and mortality rate of adult female *Ae. aegypti* against various insecticides and synergists. **Table S2.** Knockdown times KT_50_ and KT_99_ of adult female *Ae. aegypti* to various insecticides. **Table S3.** Frequency of the F1534C mutation in the *Ae. aegypti* voltage-gated sodium channel gene within resistance and susceptible mosquitoes from nine different districts of Selangor determined using AS-PCR. **Table S4.** Frequency of the V1016G mutation in the *Ae. aegypti* voltage-gated sodium channel gene within resistance and susceptible mosquitoes from nine different districts of Selangor determined using AS-PCR. **Table S5.** Frequency of the S989P mutation in the *Ae. aegypti* voltage-gated sodium channel gene within resistance and susceptible mosquitoes from nine different districts of Selangor determined using AS-PCR.

**Additional file 2: Figure S1.** Gel electrophoresis of AS-PCR products corresponding to the *Ae. aegypti* sodium channel gene mutation. **a** F1534C mutation: each of the three genotypes is shown. Lane 1: ultra-low range DNA ladder; Lane 2: wild-type homozygous (FF); Lane 3: heterozygous (FC); Lane 4: mutant homozygous (CC); Lane 5: negative control. **b** V1016G mutation: Lane 1: ultra-low range DNA ladder marker; Lane 2: mutant homozygous (GG); Lane 3: heterozygous (VG); Lane 4: wild-type homozygous (VV); Lane 5: negative control. **c** S989P mutation: Lane 1: 100 bp DNA ladder marker; Lanes 2, 3: wild-type homozygous (SS); Lanes 4, 5: heterozygous (SP); Lanes 6, 7: mutant homozygous (PP); Lanes 8, 9: negative control. **Figure S2.** Genotype sequence of V1016G mutation. **Figure S3.** Genotype sequence of S989P mutation. **Figure S4.** Genotype sequence of F1534C mutation.

## Abbreviations

AS-PCR: Allele specific polymerase chain reaction
MFO: mixed function oxidase
EST: esterase
GST: glutathione S-transferase
AcHE: acetylcholinesterase
ULV: ultra-low volume
CDC: Centre for Diseases Control and Prevention
*Kdr*: knock-down resistance
DDT: dichlorodiphenyltrichloroethane
GDP: gross domestic product
EA: ethacrynic acid
PBO: piperonyl butoxide
DEF: S.S.S-tributyl phosphorotrithioate
OD: optical density
CDNB: 1-chloro-2,4-dinitrobenzene
TMBZ: tetramethylbenzidine
RR_50_: resistance ratio 50%
