# Supplementary material for: Enzymatic and molecular characterization of insecticide resistance mechanisms in field populations of Aedes aegypti from Selangor, Malaysia
Source: Parasit Vectors. 2019 May 16;12:236. doi: 10.1186/s13071-019-3472-1 (PMC6521414; doi:10.1186/s13071-019-3472-1)
Supplement: Supplementary file 1 — Additional file 1: Table S1. Knockdown rate and mortality rate of adult female Ae. aegypti against various insecticides and synergists. Table S2. Knockdown times KT50 and KT99 of adult female Ae. aegypti to various insecticides. Table S3. Frequency of the F1534C mutation in the Ae. aegypti voltage-gated sodium channel gene within resistance and susceptible mosquitoes from nine different districts of Selangor determined using AS-PCR. Table S4. Frequency of the V1016G mutation in the Ae. aegypti voltage-gated sodium channel gene within resistance and susceptible mosquitoes from nine different districts of Selangor determined using AS-PCR. Table S5. Frequency of the S989P mutation in the Ae. aegypti voltage-gated sodium channel gene within resistance and susceptible mosquitoes from nine different districts of Selangor determined using AS-PCR. [file 13071_2019_3472_MOESM1_ESM.docx]

**Additional file 1**

**Additional file 1: Table S1:** Knockdown rate and mortality rate of adult female *Aedes aegypti* against various insecticides and synergists.

| Stain | Bora-Bora | | G | | HL | | HS | | K | | KL | | KS | | P | | SB | | S | |
| --- | --- | --- | --- | --- | --- | --- | --- | --- | --- | --- | --- | --- | --- | --- | --- | --- | --- | --- | --- | --- |
| Insecticides | D | 24h | D | 24h | D | 24h | D | 24h | D | 24h | D | 24h | D | 24h | D | 24h | D | 24h | D | 24h |
| Total exposed n | 908 | | 905 | | 905 | | 914 | | 911 | | 924 | | 907 | | 909 | | 920 | | 916 | |
| Malathion  only | 100 ± 0 | 100 ± 0* | 100 ± 0* | 100 ± 0 | 93.55 ± 1.39 | 100 ± 0* | 94.03 ± 2.59 | 100 ± 0* | 100 ± 0 | 100 ± 0* | 100 ± 0 | 100 ± 0* | 100 ± 0 | 100 ± 0* | 84.02 ± 7.29 | 100 ± 0* | 93.03 ± 3.49 | 100 ± 0* | 97.37 ± 2.63 | 100 ± 0* |
| Malathion  + EA | 100 ± 0 | 100 ± 0* | 100 ± 0* | 100 ± 0 | 100 | 100 ± 0* | 100 | 100 ± 0* | 100 ± 0 | 100 ± 0* | 100 ± 0 | 100 ± 0* | 100 ± 0 | 100 ± 0* | 96.47 ± 1.91 | 100 ± 0* | 97.47 ± 2.53 | 100 ± 0* | 100 ± 0 | 100 ± 0* |
| Malathion  + DEF | 100 ± 0 | 100 ± 0* | 100 ± 0* | 100 ± 0 | 100 | 100 ± 0* | 100 | 100 ± 0* | 100 ± 0 | 100 ± 0* | 100 ± 0 | 100 ± 0* | 100 ± 0 | 100 ± 0* | 99.12 ± 0.88 | 100 ± 0* | 100 ± 0 | 100 ± 0* | 100 ± 0 | 100 ± 0* |
| Malathion  + PBO | 100 ± 0 | 100 ± 0* | 100 ± 0* | 100 ± 0 | 100 | 100 ± 0* | 100 | 100 ± 0* | 100 ± 0 | 100 ± 0* | 100 ± 0 | 100 ± 0* | 100 ± 0 | 100 ± 0* | 100 ± 0 | 100 ± 0* | 91.75 ± 6.91 | 100 ± 0* | 100 ± 0 | 100 ± 0* |
| Total exposed n | 894 | | 909 | | 918 | | 902 | | 908 | | 917 | | 914 | | 904 | | 909 | | 909 | |
| DDT  only | 100 ± 0 | 100 ± 0* | 24.62 ± 3.25^#^ | 69.64 ± 2.82^#^ | 1.25 ± 1.25^#^ | 22.26 ± 3.99^#^ | 0 | 10.69 ± 1.41^#^ | 100 ± 0 | 100 ± 0* | 4.83 ± 1.19^#^ | 37.07 ± 1.74^#^ | 5.64 ± 1.05^#^ | 41.63 ± 8.30^#^ | 6.77 ± 1.07^#^ | 22.60 ± 6.92^#^ | 44.31 ± 10.12^#^ | 100 ± 0* | 16.62 ± 1.05^#^ | 87.67 ± 1.20^#^ |
| DDT  + EA | 100 ± 0 | 100 ± 0* | 18.42 ± 2.01^#^ | 75.88 ± 2.88^#^ | 20.86 ± 1.43^#a^ | 65.64 ± 2.25^#a^ | 2.67 ± 0^#^ | 16.00 ± 1.33^#^ | 100 ± 0 | 100 ± 0* | 10.19 ± 1.16^#^ | 35.69 ± 1.40^#^ | 9.69 ± 0.84^#^ | 30.38 ± 1.41^#^ | 20.00 ± 1.54^#^ | 73.78 ± 2.35^#a^ | 70.07 ± 6.13^#a^ | 100 ± 0* | 19.01 ± 1.85^#^ | 91.60 ± 1.57^#^ |
| DDT  + DEF | 100 ± 0 | 100 ± 0* | 25.99 ± 0.39^#^ | 87.67 ± 1.58^#^ | 23.24 ± 2.60^#a^ | 75.89 ± 2.31^#a^ | 9.78 ± 0.89^#^ | 37.33 ± 2.78^#a^ | 100 ± 0 | 100 ± 0* | 8.53 ± 0.32^#^ | 41.02 ± 1.10^#^ | 8.83 ± 1.23^#^ | 33.94 ± 1.80^#^ | 28.00 ± 2.31^#a^ | 85.33 ± 5.39^a^ | 67.56 ± 2.35^#a^ | 100 ± 0* | 26.08 ± 3.36^#^ | 90.28 ± 2.31 |
| DDT  + PBO | 100 ± 0 | 100 ± 0* | 27.28 ± 3.81^#^ | 88.07 ± 3.56 | 24.71 ± 3.40^#a^ | 82.38 ± 0.82^#a^ | 11.87 ± 1.29^#^ | 55.53 ± 2.27^#a^ | 100 ± 0 | 100 ± 0* | 18.82 ± 2.46^#a^ | 89.44 ± 3.57^a^ | 9.09 ± 0.12^#^ | 64.76 ± 6.90^#a^ | 38.41 ± 3.43^#a^ | 92.95 ± 2.92^a^ | 58.93 ± 9.33^#^ | 100 ± 0* | 25.04 ± 2.94^#^ | 89.40 ± 5.42 |
| Total exposed n | 912 | | 909 | | 913 | | 914 | | 903 | | 926 | | 913 | | 905 | | 909 | | 912 | |
| Propoxur  only | 100 ± 0 | 100 ± 0* | 100 ± 0 | 100 ± 0* | 100 ± 0 | 100 ± 0* | 100 ± 0 | 100 ± 0* | 100 ± 0 | 100 ± 0* | 100 ± 0 | 100 ± 0* | 100 ± 0 | 100 ± 0* | 55.57 ± 15.95^#^ | 92.22 ± 2.66 | 40.61 ± 5.76^#^ | 100 ± 0* | 91.75 ± 2.35^#^ | 100 ± 0* |
| Propoxur  + EA | 100 ± 0 | 100 ± 0* | 100 ± 0 | 100 ± 0* | 100 ± 0 | 100 ± 0* | 100 ± 0 | 100 ± 0* | 100 ± 0 | 100 ± 0* | 100 ± 0 | 100 ± 0* | 100 ± 0 | 100 ± 0* | 100 ± 0^a^ | 100 ± 0* | 41.06 ± 5.30^#^ | 100 ± 0* | 100 ± 0 | 100 ± 0* |
| Propoxur  + DEF | 100 ± 0 | 100 ± 0* | 100 ± 0 | 100 ± 0* | 100 ± 0 | 100 ± 0* | 100 ± 0 | 100 ± 0* | 100 ± 0 | 100 ± 0* | 100 ± 0 | 100 ± 0* | 100 ± 0 | 100 ± 0* | 100 ± 0^a^ | 100 ± 0* | 98.24 ± 0.88^a^ | 100 ± 0* | 100 ± 0 | 100 ± 0* |
| Propoxur  + PBO | 100 ± 0 | 100 ± 0* | 100 ± 0 | 100 ± 0* | 100 ± 0 | 100 ± 0* | 100 ± 0 | 100 ± 0* | 100 ± 0 | 100 ± 0* | 100 ± 0 | 100 ± 0* | 100 ± 0 | 100 ± 0* | 100 ± 0^a^ | 100 ± 0* | 53.51 ± 3.53^#^ | 100 ± 0* | 97.81 ± 2.19 | 100 ± 0* |
| Total exposed n | 897 | | 905 | | 905 | | 931 | | 909 | | 920 | | 907 | | 886 | | 918 | | 915 | |
| Cyfluthrin  only | 100 ± 0 | 100 ± 0* | 26.11 ± 0.40^#^ | 100 ± 0 | 18.14 ± 041^#^ | 77.89 ± 3.06 | 3.11 ± 0.44^#^ | 12.00 ± 3.85^#^ | 100 ± 0 | 100 ± 0* | 45.35 ± 16.29^#^ | 100 ± 0* | 5.70 ± 0.40^#^ | 28.09 ± 6.05^#^ | 10.00 ± 0.14^#^ | 21.86 ± 1.57^#^ | 77.14 ± 7.89 | 100 ± 0* | 20.99 ± 2.48^#^ | 92.93 ± 1.46 |
| Cyfluthrin  + EA | 100 ± 0 | 100 ± 0* | 60.30 ± 7.43^#^ | 100 ± 0* | 5.70 ± 0.44^#^ | 35.53 ± 1.52^#^ | 8.44 ± 0.44^#a^ | 35.56 ± 0.44^#a^ | 100 ± 0 | 100 ± 0* | 41.98 ± 8.90^#^ | 100 ± 0* | 5.29 ± 0.76^#^ | 35.65 ± 4.45^#^ | 8.89 ± 0.44^#^ | 50.66 ± 4.81^#a^ | 100 ± 0^a^ | 100 ± 0* | 36.72 ± 4.24^#^ | 100 ± 0 |
| Cyfluthrin  + DEF | 100 ± 0 | 100 ± 0* | 33.00 ± 2.46^#a^ | 100 ± 0 | 14.67 ± 0.00^#^ | 52.89 ± 1.18^#^ | 8.00 ± 0.00^#a^ | 45.33 ± 6.11^#a^ | 100 ± 0 | 100 ± 0* | 52.31 ± 7.67^#^ | 100 ± 0* | 3.51 ± 1.58^#^ | 25.52 ± 6.33^#^ | 30.54 ± 2.12^#a^ | 73.04 ± 2.96^#a^ | 94.72 ± 0.80^a^ | 100 ± 0* | 31.25 ± 2.96^#^ | 92.95 ± 0.42 |
| Cyfluthrin  + PBO | 100 ± 0 | 100 ± 0* | 50.67 ± 4.29^#^ | 100 ± 0 | 41.27 ± 18.83^#a^ | 67.45 ± 17.43^#^ | 16.82 ± 0.51^#a^ | 45.60 ± 3.90^#a^ | 100 ± 0 | 100 ± 0* | 100 ± 0^a^ | 100 ± 0* | 12.89 ± 1.78^#^ | 54.67 ± 1.54^#a^ | 31.11 ± 1.18^#a^ | 100 ± 0*^a^ | 100 ± 0^a^ | 100 ± 0* | 41.21 ± 1.29^#a^ | 100 ± 0* |
| Total exposed n | 897 | | 906 | | 905 | | 901 | | 908 | | 919 | | 917 | | 899 | | 919 | | 916 | |
| Deltamethrin  only | 100 ± 0 | 100 ± 0* | 20.35 ± 0.84^#^ | 76.56 ± 1.10^#^ | 41.59 ± 29.27 | 78.54 ± 16.48 | 6.64 ± 0.77^#^ | 30.98 ± 2.21^#^ | 100 ± 0 | 100 ± 0* | 45.59 ± 13.20 | 100 ± 0* | 5.80 ± 2.01^#^ | 30.01 ± 3.22^#^ | 2.74 ± 0.02^#^ | 18.25 ± 1.06^#^ | 66.85 ± 8.38 | 100 ± 0* | 15.75 ± 0.76^#^ | 99.82 ± 3.47 |
| Deltamethrin  + EA | 100 ± 0 | 100 ± 0* | 18.09 ± 2.98^#^ | 83.70 ± 3.42^#^ | 3.98 ± 0.02^#^ | 34.07 ± 0.97^#^ | 8.41 ± 0.46^#^ | 41.60 ± 2.73^#^ | 100 ± 0 | 100 ± 0* | 55.43 ± 4.77^#^ | 100 ± 0* | 3.95 ± 1.50^#^ | 29.77 ± 3.71^#^ | 15.04 ± 0.86^#a^ | 71.68 ± 0.83^#a^ | 76.63 ± 11.37 | 100 ± 0* | 28.76 ± 1.16^#a^ | 100 ± 0* |
| Deltamethrin  + DEF | 100 ± 0 | 100 ± 0* | 17.98 ± 1.12^#^ | 84.66 ± 2.30^#^ | 9.33 ± 0.07^a^ | 43.10 ± 0.99^a^ | 6.72 ± 1.54^#^ | 54.22 ± 4.29^#^ | 100 ± 0 | 100 ± 0* | 88.47 ± 7.84^a^ | 100 ± 0* | 4.44 ± 0.44^#^ | 26.22 ± 3.95^#^ | 19.75 ± 0.91^#a^ | 81.56 ± 1.66^#a^ | 88.16 ± 1.49^#a^ | 100 ± 0* | 25.65 ± 1.14^#^ | 96.08 ± 0.78 |
| Deltamethrin  + PBO | 100 ± 0 | 100 ± 0* | 30.67 ± 7.34^#^ | 100 ± 0*^a^ | 18.50 ± 1.52^#^ | 76.70 ± 6.59 | 11.44 ± 1.55^#^ | 65.23 ± 4.55^#^ | 100 ± 0 | 100 ± 0* | 84.58 ± 6.91^a^ | 100 ± 0* | 8.28 ± 1.20^#^ | 51.75 ± 3.16^#a^ | 42.03 ± 3.72^#a^ | 100 ± 0*^a^ | 97.80 ± 0.45^a^ | 100 ± 0* | 30.67 ± 1.54^#a^ | 100 ± 0* |
| Total exposed n | 911 | | 907 | | 899 | | 900 | | 908 | | 916 | | 909 | | 891 | | 906 | | 913 | |
| Etofenprox  only | 100 ± 0 | 100 ± 0* | 30.02 ± 7.80^#^ | 79.76 ± 2.81^#^ | 10.92 ± 6.04 | 54.05 ± 2.15 | 4.47 ± 0.93^#^ | 47.58 ± 3.22^#^ | 99.56 ± 0.44 | 100 ± 0* | 2.23 ± 1.18^#^ | 38.08 ± 3.60^#^ | 2.28 ± 1.40^#^ | 34.27 ± 3.08 | 2.40 ± 1.04^#^ | 25.70 ± 0.90^#^ | 34.85 ± 9.53^#^ | 100 ± 0* | 19.22 ± 2.47^#^ | 86.74 ± 4.34^#^ |
| Etofenprox  + EA | 100 ± 0 | 100 ± 0* | 16.67 ± 1.24^#^ | 83.72 ± 3.59^#^ | 30.09 ± 1.16^#^ | 61.51 ± 1.39^#^ | 7.56 ± 0.44^#^ | 45.78 ± 3.08^#^ | 100 ± 0 | 100 ± 0* | 14.91 ± 1.16^#a^ | 69.30 ± 3.75^#a^ | 7.93 ± 0.79^#^ | 37.02 ± 1.67^#^ | 9.78 ± 1.18^#^ | 52.89 ± 3.47^#a^ | 71.23 ± 13.56^a^ | 100 ± 0* | 20.71 ± 0.49^#^ | 92.08 ± 1.99 |
| Etofenprox  + DEF | 100 ± 0 | 100 ± 0* | 23.90 ± 0.86^#^ | 91.58 ± 1.79^a^ | 12.89 ± 0.44^#^ | 51.55 ± 1.78^#^ | 15.11 ± 3.80^#a^ | 58.22 ± 2.35^#^ | 100 ± 0 | 100 ± 0* | 11.62 ± 0.42^#a^ | 72.82 ± 0.62^#a^ | 6.67 ± 0.77^#^ | 15.11 ± 1.18^#^ | 24.35 ± 1.83^#a^ | 62.41 ± 4.37^a^ | 57.01 ± 1.20^#a^ | 100 ± 0* | 19.55 ± 3.21^#^ | 92.00 ± 2.67 |
| Etofenprox  + PBO | 100 ± 0 | 100 ± 0* | 31.40 ± 1.46^#^ | 100 ± 0^a^ | 31.73 ± 3.73^#^ | 85.51 ± 3.40^a^ | 19.55 ± 1.18^#a^ | 74.22 ± 3.95^#a^ | 100 ± 0 | 100 ± 0* | 21.74 ± 2.64^#a^ | 93.47 ± 1.99^a^ | 10.96 ± 2.71^#^ | 76.74 ± 5.24^#a^ | 72.52 ± 5.31^#a^ | 96.32 ± 2.67*^a^ | 69.78 ± 12.80^a^ | 100 ± 0* | 21.67 ± 2.17^#^ | 92.46 ± 1.95 |
| Total exposed n | 909 | | 906 | | 916 | | 932 | | 913 | | 915 | | 921 | | 897 | | 920 | | 912 | |
| Lambdacyhalothrin only | 100 ± 0 | 100 ± 0* | 27.47 ± 4.59^#^ | 83.39 ± 1.63^#^ | 11.11 ± 2.35^#^ | 29.33 ± 2.83^#^ | 11.11 ± 1.60^#^ | 45.67 ± 3.79^#^ | 100 ± 0 | 100 ± 0* | 27.32 ± 1.88^#^ | 68.13 ± 9.12^#^ | 11.57 ± 5.84^#^ | 64.59 ± 17.49 | 4.55 ± 0.58^#^ | 35.10 ± 5.31^#^ | 62.35 ± 10.71 | 100 ± 0* | 15.15 ± 0.33^#^ | 82.29 ± 0.67^#^ |
| Lambdacyhalothrin  + EA | 100 ± 0 | 100 ± 0* | 17.71 ± 1.23^#^ | 84.48 ± 5.15 | 32.64 ± 7.07^#a^ | 72.16 ± 10.47*^a^ | 23.56 ± 1.94^#^ | 73.62 ± 6.67^#^ | 100 ± 0 | 100 ± 0* | 27.19 ± 1.16^#^ | 74.41 ± 6.41^#^ | 22.81 ± 4.39^#^ | 83.33 ± 2.44^#^ | 14.58 ± 2.69^#a^ | 80.12 ± 4.17^a^ | 56.52 ± 11.49^#^ | 100 ± 0* | 42.31 ± 3.10^#a^ | 100 ± 0* |
| Lambdacyhalothrin  + DEF | 100 ± 0 | 100 ± 0* | 22.13 ± 0.94^#^ | 92.48 ± 2.46 | 12.44 ± 0.44^#^ | 49.78 ± 1.60^#a^ | 27.72 ± 5.97^#^ | 66.67± 9.38^#^ | 100 ± 0 | 100 ± 0* | 31.26 ± 0.81^#^ | 88.14 ± 7.47^#^ | 8.76 ± 0.82^#^ | 42.47 ± 3.74^#^ | 28.89 ± 1.18^#a^ | 100 ± 0*^a^ | 90.36 ± 3.91^a^ | 100 ± 0* | 21.51 ± 1.69^#^ | 94.25 ± 3.11 |
| Lambdacyhalothrin  + PBO | 100 ± 0 | 100 ± 0* | 27.56 ± 0.44^#^ | 99.56 ± 0.44^a^ | 42.68± 6.33^#a^ | 89.31 ± 11.74*^a^ | 34.22 ± 5.57^#^ | 89.12 ± 10.98 | 100 ± 0 | 100 ± 0* | 44.33 ± 2.51^#^ | 91.36 ± 13.73 | 31.44 ± 6.48^#a^ | 82.34 ± 8.99^a^ | 71.63 ± 6.34^#a^ | 100 ± 0*^a^ | 100 ± 0^a^ | 100 ± 0* | 21.68 ± 3.10^#^ | 100 ± 0* |
| Total exposed n | 889 | | 905 | | 906 | | 906 | | 911 | | 921 | | 907 | | 917 | | 912 | | 910 | |
| Permethrin  only | 100 ± 0 | 100 ± 0* | 19.84 ± 1.61^#^ | 73.53 ± 4.37^#^ | 17.36 ± 6.96^#^ | 50.25 ± 12.09 | 0 | 14.46 ± 1.72^#^ | 100 ± 0 | 100 ± 0* | 2.65 ± 1.33^#^ | 40.07 ± 2.70^#^ | 0.88 ± 0.44^#^ | 46.49 ± 4.18^#^ | 4.93 ± 1.085^#^ | 40.54 ± 22.87^#^ | 43.34 ± 6.15^#^ | 100 ± 0* | 10.70 ± 1.46^#^ | 92.71 ± 2.06 |
| Permethrin  + EA | 100 ± 0 | 100 ± 0* | 14.13 ± 1.83^#^ | 82.83 ± 5.68 | 16.00 ± 0.00^#^ | 57.33 ± 2.04^a^ | 24.44 ± 0.89^#a^ | 28.89 ± 0.44^#a^ | 100 ± 0 | 100 ± 0* | 7.96 ± 0.88^#^ | 41.91 ± 1.81^#^ | 4.44 ± 0.41^#a^ | 33.81 ± 2.51^#^ | 5.75 ± 0.46^#^ | 59.33 ± 4.02^#a^ | 49.54 ± 6.91^#^ | 100 ± 0* | 16.00 ± 3.85^#^ | 100.00 ± 0 |
| Permethrin  +DEF | 100 ± 0 | 100 ± 0* | 19.49 ± 2.40^#^ | 84.96 ± 1.59^#^ | 12.00 ± 1.33^#^ | 56.44 ± 5.24^#^ | 8.94 ± 1.21^#a^ | 52.66 ± 2.15^#a^ | 100 ± 0 | 100 ± 0* | 5.29 ± 2.04^#^ | 75.89 ± 1.86^#a^ | 5.32 ± 1.31^#a^ | 27.08 ± 2.77^#^ | 24.89 ± 1.60^#a^ | 98.67 ± 0.77^a^ | 39.67 ± 2.20^#^ | 100 ± 0* | 14.99 ± 3.55^#^ | 92.50 ± 2.47 |
| Permethrin  + PBO | 100 ± 0 | 100 ± 0* | 25.19 ± 5.30^#^ | 89.36 ± 3.37^a^ | 26.98 ± 1.52^#^ | 89.41 ± 4.23^a^ | 9.22 ± 0.81^#a^ | 58.33 ± 0.55^#a^ | 100 ± 0 | 100 ± 0* | 18.48 ± 3.44^#a^ | 81.49 ± 1.60^#a^ | 8.30 ± 0.91^#a^ | 60.37 ± 1.66^#a^ | 81.80 ± 8.53^a^ | 100 ± 0*^a^ | 96.91 ± 1.91^a^ | 100 ± 0* | 20.83 ± 4.38^#a^ | 100 ± 0 |

D- Diagnostic time, 24h= 24 hours post-treatment.

Mean % mortality followed by asterisk symbol denotes 100% mortality was achieved within 2 hours exposure.

Mean % mortality followed by number sign denotes rates that were significantly different when compared with to Bora-bora strain (*P* < 0.05, independent *T*-test).

Mean followed by a superscript letter is a mean with significant difference between synergist-treated versus non-synergist-treated, (*P <* 0.05, Mann-Whitney U test).

**Additional file 1: Table S2.** Knockdown times KT_50_ and KT_99_ of adult female *Aedes aegypti* to various insecticides.

| Insecticide | Strain | n | KT_50_ (95% CL) (min) | KT_99_ (95% CL) (min) | Slope | χ2 (df) |  |
| --- | --- | --- | --- | --- | --- | --- | --- |
| Malathion | Bora-Bora | 227 | 22.95 (22.30 - 23.65) | 42.81 (39.25 – 48.00) | 32.72 ± 11.69 | 72.20 (15) |  |
|  | G | 228 | 14.82 (14.56 – 15.09) | 39.83 (37.97 – 42.00) | 36.85 ± 6.35 | 11.60 (19) |  |
|  | HL | 234 | 24.13 (23.22 – 25.11) | 52.23 (46.89 – 60.25) | 41.02 ± 9.59 | 184.26 (22) |  |
|  | HS | 233 | 20.82 (20.24 – 21.41) | 38.83 (36.23 – 42.35) | 39.72 ± 11.33 | 91.172 (18) |  |
|  | K | 227 | 13.70 (13.32 – 14.09) | 36.08 (33.37 – 39.54) | 34.53 ± 6.29 | 31.40 (16) |  |
|  | KL | 230 | 15.47 (14.61 – 16.42) | 40.15 (34.19 – 50.42) | 34.32 ± 6.68 | 133.91 (16) |  |
|  | KS | 228 | 17.76 (17.27 – 18.25) | 42.96 (39.75 – 47.11) | 37.78 ± 7.57 | 51.67 (19) |  |
|  | P | 227 | 28.50 (28.25 – 28.76) | 46.81 (45.75 – 47.99) | 40.56 ± 15.71 | 7.80 (20) |  |
|  | SB | 232 | 21.02 (20.41 – 21.65) | 67.02 (61.10 – 74.69) | 40.82 ± 6.11 | 70.17 (26) |  |
|  | S | 232 | 15.17 (14.86 – 15.47) | 52.21 (49.45 – 55.41) | 39.52 ± 5.12 | 18.98 (24) |  |
| DDT | Bora-Bora | 212 | 27.18 (26.39 – 28.00) | 65.79 (60.07 – 73.53) | 40.148 ± 8.69 | 108.53 (26) |  |
|  | G | 227 | 85.30 (83.82 – 86.85) | 660.88 (609.63 – 720.67) | 57.45 ± 5.05 | 27.20 (97) |  |
|  | HL | 237 | 455.54 (338.18 – 724.79) | 7869.14 (3502.17 – 27870.99) | 12.19 ± 5.00 | 1.89 (51) |  |
|  | HS | 226 | N.A. | N.A. | N.A. | N.A. |  |
|  | K | 228 | 21.48 (21.10 – 21.85) | 50.02 (47.65 – 52.80) | 41.10 ± 8.44 | 32.99 (23) |  |
|  | KL | 229 | 201.26 (186.52 – 220.25) | 1853.27 (1437.89 – 2508.36) | 26.73 ± 5.56 | 6.54 (69) |  |
|  | KS | 229 | 251.40 (227.65 – 283.01) | 2970.54 (2195.50 – 4267.90) | 27.31 ± 5.21 | 12.26 (74) |  |
|  | P | 225 | 505.44 (389.46 – 720.47) | 19614.88 (9293.40 – 54318.40) | 18.41 ± 3.96 | 2.67 (66) |  |
|  | SB | 229 | 43.93 (42.99 – 48.87) | 210.45 (195.47 – 228.21) | 61.40 ± 5.62 | 160.97 (71) |  |
|  | S | 229 | 103.32 (101.14 – 105.68) | 727.03 (660.40 – 807.19) | 49.49 ± 5.53 | 8.23(88) |  |
| Propoxur | Bora-Bora | 218 | 29.59 (28.74 – 30.51) | 65.157 (59.72 – 72.99) | 40.11 ± 9.98 | 119.90 (25) |  |
|  | G | 227 | 18.22 (17.21 – 19.28) | 91.39 (76.63 – 114.07) | 40.70 ± 4.19 | 66.61 (30) |  |
|  | HL | 240 | 9.42 (9.26 – 9.58) | 18.01 (17.34 – 18.81) | 29.43 ± 8.05 | 8.65 (9) |  |
|  | HS | 235 | 16.60 (16.31 – 16.89) | 47.92 (45.66 – 50.54) | 38.44 ± 6.16 | 10.908 (22) |  |
|  | K | 227 | 10.67 (10.09 – 11.26) | 36.10 (31.17 – 43.66) | 31.95 ± 4.52 | 65.77 (15) |  |
|  | KL | 232 | 11.61 (11.09 – 12.15) | 36.54 (32.31 – 42.60) | 34.26 ± 4.98 | 59.54 (16) |  |
|  | KS | 231 | 9.74 (9.55 – 9.93) | 22.39 (21.25 – 23.75) | 29.17 ± 6.36 | 10.13 (10) |  |
|  | P | 223 | 46.35 (45.26 – 47.40) | 199.42 (188.26 – 212.36) | 64.58 ± 6.12 | 243.55 (92) |  |
|  | SB | 228 | 47.69 (46.50 – 48.91) | 183.86 (169.27 – 201.79) | 63.71 ± 6.66 | 267.38 (69) |  |
|  | S | 228 | 21.01 (20.10 – 21.94) | 109.59 (96.62 – 127.04) | 48.40 ± 4.29 | 189.50 (42) |  |
| Cyfluthrin | Bora-Bora | 220 | 31.52 (30.54 – 32.60) | 66.61 (60.22 – 75.96) | 38.71 ± 10.729 | 154.47 (24) |  |
|  | G | 227 | 36.01 (35.29 – 36.73) | 145.12 (136.37 – 155.33) | 57.48 ± 5.98 | 109.47 (58) |  |
|  | HL | 226 | 99.20 (96.37 – 102.32) | 777.87 (687.06 – 892.85) | 58.67 ± 5.19 | 215.58 (101) |  |
|  | HS | 225 | 640.53 (369.39 – 2417.66) | 14280.37 (3318.17 – 485794.00) | 6.65 ± 4.84 | 2.32 (39) |  |
|  | K | 228 | 14.91 (14.22 – 15.63) | 57.04 (49.64 – 104.09) | 36.88 ± 4.67 | 93.20 (22) |  |
|  | KL | 232 | 44.87 (43.92 – 45.82) | 250.71 (193.58 – 219.72) | 72.75 ± 5.81 | 232.60 (89) |  |
|  | KS | 228 | 837.43 (770.50 – 4780.53) | 5731.89 (1666.71 – 7620.10) | 10.84 ± 2.37 | 4.62 (71) |  |
|  | P | 210 | 1925.08 (1100.77 – 4450.68) | 1.51 x 10^6^ (2.84 x 10^5^ – 1.87 x 10^7^) | 17.34 ± 2.64 | 4.44 (79) |  |
|  | SB | 292 | 38.23 (37.66 – 38.82) | 149.98 (140.98 – 160.42) | 47.50 ± 6.20 | 10.13 (39) |  |
|  | S | 237 | 86.26 (84.77 – 87.83) | 627.06 (576.82 – 686.16) | 66.46 ± 5.79 | 40.22 (90) |  |
| Deltamethrin | Bora-Bora | 217 | 24.01 (23.37 – 24.69) | 47.06 (43.01 – 52.12) | 35.31 ± 10.98 | 72.68 (18) |  |
|  | G | 226 | 83.66 (82.03 – 85.38) | 861.80 (787.26 – 949.53) | 59.19 ± 4.42 | 107.81 (102) |  |
|  | HL | 227 | 95.10 (92.26 – 98.20) | 1243.49 (1084.22 – 1445.48) | 58.43 ± 4.12 | 174.71 (106) |  |
|  | HS | 226 | 296.87 (259.18 – 350.61) | 6694.94 (4376.82 – 11290.74) | 25.20 ± 4.25 | 15.02 (73) |  |
|  | K | 229 | 13.75 (13.20 – 14.33) | 39.40 (34.88 – 45.99) | 32.02 ± 5.79 | 52.11 (15) |  |
|  | KL | 227 | 36.64 (36.06 – 37.21) | 137.41 (131.54 – 143.97) | 63.21 ± 6.34 | 86.12 (65) |  |
|  | KS | 236 | 5984.50 (2292.42 – 31419.83) | 1.12 x 10^7^ (8.74 x 10^5^ – 9.16 x 10^8^) | 15.24 ± 2.69 | 1.56 (70) |  |
|  | P | 219 | 269.58 (222.34 – 364.88) | 1694.52 (999.83 – 3890.66) | 10.77 ± 7.08 | 1.66 (40) |  |
|  | SB | 232 | 28.51 (27.92 – 29.12) | 74.67 (69.97 – 80.42) | 45.58 ± 8.10 | 73.35 (31) |  |
|  | S | 235 | 78.90 (77.64 – 80.21) | 615.74 (570.54 – 668.12) | 58.50 ± 4.95 | 39.61 (97) |  |
| Etofenprox | Bora-Bora | 232 | 19.38 (18.91 – 19.87) | 44.15 (41.04 – 48.18) | 36.30 ± 8.37 | 45.32 (18) |  |
|  | G | 227 | 66.81 (65.62 – 68.03) | 769.28 (705.21 – 844.32) | 57.60 ± 4.00 | 23.66 (102) |  |
|  | HL | 224 | 151.31 (144.13 – 162.52) | 902.49 (871.11 – 939.48) | 70.04 ± 4.45 | 251.66 (109) |  |
|  | HS | 225 | 215.19 (200.59 – 235.82) | 2218.97 (1744.98 – 2937.64) | 30.92 ± 5.37 | 35.92 (78) |  |
|  | K | 228 | 15.24 (14.63 – 15.87) | 51.78 (45.88 – 60.07) | 35.54 ± 5.18 | 68.55 (20) |  |
|  | KL | 226 | 174.52 (165.34 – 185.78) | 1105.55 (920.08 – 1369.44) | 29.56 ± 6.51 | 9.42 (69) |  |
|  | KS | 226 | 153.73 (132.49 –164.99) | 1284.73 (1162.49 – 1311.42) | 66.91 ± 5.56 | 264.48 (90) |  |
|  | P | 214 | 306.01 (263.42 – 371.21) | 3959.50 (2552.58 – 6986.50) | 19.73 ± 5.20 | 4.04 (65) |  |
|  | SB | 226 | 46.59 (45.42 – 47.77) | 229.67 (209.73 – 254.30) | 57.15 ± 5.60 | 213.72 (71) |  |
|  | S | 235 | 129.11 (123.86 – 135.03) | 4071.49 (3366.93 – 5015.85) | 48.84 ± 3.28 | 11.56 (104) |  |
| Lambdacyhalothrin | Bora-Bora | 230 | 21.28 (20.74 – 21.85) | 45.26 (41.78 – 49.91) | 34.79 ± 9.42 | 50.57 (17) |  |
|  | G | 229 | 62.60 (61.64 – 63.57) | 532.66 (498.45 – 571.59) | 63.01 ± 4.49 | 21.26 (104) |  |
|  | HL | 225 | 128.37 (124.38 – 132.80) | 1374.10 (1207.29 – 1583.03) | 49.17 ± 4.76 | 75.25 (97) |  |
|  | HS | 255 | 64.18 (60.94 – 68.03) | 221.41 (185.30 – 279.79) | 60.15 ± 7.82 | 1128.92 (68) |  |
|  | K | 228 | 18.01 (17.13 – 18.99) | 51.17 (43.71 – 63.35) | 34.33 ± 6.44 | 113.24 (18) |  |
|  | KL | 231 | 48.63 (47.49 – 49.81) | 198.26 (183.08 – 216.69) | 64.22 ± 6.43 | 248.40 (69) |  |
|  | KS | 237 | 106.35 (103.92 – 108.98) | 970.27 (876.11 – 1083.55) | 54.01 ± 4.91 | 16.93 (97) |  |
|  | P | 221 | 255.46 (232.72 – 285.21) | 2992.48 (2249.14 – 4199.93) | 28.70 ± 5.24 | 17.44 (79) |  |
|  | SB | 231 | 28.05 (27.53 – 28.56) | 90.96 (86.01 – 96.73) | 49.55 ± 6.59 | 59.25 (39) |  |
|  | S | 231 | 81.77 (80.47 – 83.13) | 596.65 (554.83 – 644.80) | 60.66 ± 5.16 | 21.29 (99) |  |
| Permethrin | Bora-Bora | 212 | 22.30 (21.40 – 23.32) | 48.10 (42.47 – 57.11) | 34.24 ± 9.40 | 129.85 (18) |  |
|  | G | 227 | 85.76 (84.04 – 87.59) | 903.28 (819.33 – 10003.35) | 55.22 ± 4.40 | 15.42 (99) |  |
|  | HL | 230 | 70.96 (67.70 – 74.65) | 2525.21 (1916.63 – 3478.83) | 45.23 ± 2.78 | 248.14 (95) |  |
|  | HS | 229 | 708.13 (421.92 – 2082.53) | 19061.84 (4823.54 – 335139.80) | 32.68 ± 5.20 | 30.03 (45) |  |
|  | K | 231 | 18.80 (17.53 – 20.21) | 55.61 (45.06 – 77.04) | 33.65 ± 6.30 | 245.01 (19) |  |
|  | KL | 228 | 167.88 (160.02 – 177.47) | 844.05 (717.65 – 1020.04) | 28.76 ± 7.38 | 9.62 (65) |  |
|  | KS | 228 | 241.14 (213.81 – 281.67) | 2270.86 (1559.22 – 3698.40) | 18.58 ± 5.69 | 2.96 (57) |  |
|  | P | 239 | 168.79 (158.77 – 180.74) | 7155.38 (5454.35 – 9721.84) | 39.27 ± 3.18 | 59.41 (97) |  |
|  | SB | 232 | 36.06 (35.64 – 36.49) | 102.12 (98.75 – 105.83) | 57.34 ± 8.01 | 42.12 (49) |  |
|  | S | 233 | 108.15 (105.74 – 110.77) | 838.92 (760.09 – 933.72) | 51.80 ± 5.32 | 11.61 (93) |  |

**Additional file 1: Table S3.** Frequency of the F1534C mutation in the *Ae. aegypti* voltage-gated sodium channel gene within resistance and susceptible mosquitoes from nine different districts of Selangor determined using AS-PCR.

| Strain | n | Status | FF (Homozygous) | FC (Heterozygous) | CC  (Homozygous) | Frequency of C alleles |
| --- | --- | --- | --- | --- | --- | --- |
| Bora | 30 | S | 30 | 0 | 0 | 0.00 |
| G | 30 | R | 6 | 13 | 11 | 0.61 |
|  | 30 | S | 7 | 23 | 0 | 0.38 |
| HS | 30 | R | 7 | 9 | 14 | 0.61 |
|  | 30 | S | 6 | 24 | 0 | 0.40 |
| HL | 30 | R | 6 | 9 | 15 | 0.65 |
|  | 30 | S | 21 | 9 | 0 | 0.15 |
| KL | 30 | R | 2 | 20 | 8 | 0.60 |
|  | 30 | S | 25 | 5 | 0 | 0.20 |
| KS | 30 | R | 4 | 10 | 16 | 0.70 |
|  | 30 | S | 16 | 15 | 0 | 0.25 |
| P | 30 | R | 2 | 12 | 16 | 0.73 |
|  | 30 | S | 13 | 17 | 0 | 0.31 |
| K | 30 | S | 30 | 0 | 0 | 0.00 |
| SB | 30 | S | 24 | 6 | 0 | 0.10 |
| S | 30 | R | 4 | 26 | 0 | 0.43 |
|  | 30 | S | 6 | 24 | 0 | 0.40 |

**Additional file 1: Table S4.** Frequency of the V1016G mutation in the *Ae. aegypti* voltage-gated sodium channel gene within resistance and susceptible mosquitoes from nine different districts of Selangor determined using AS-PCR.

| Strain | n | Status | VV  (Homozygous) | VG (Heterozygous) | GG  (Homozygous) | Frequency of G alleles |
| --- | --- | --- | --- | --- | --- | --- |
| Bora | 30 | S | 30 | 0 | 0 | 0.00 |
| G | 30 | R | 5 | 20 | 5 | 0.50 |
|  | 30 | S | 22 | 8 | 0 | 0.13 |
| HS | 30 | R | 2 | 16 | 12 | 0.67 |
|  | 30 | S | 3 | 27 | 0 | 0.45 |
| HL | 30 | R | 2 | 22 | 6 | 0.57 |
|  | 30 | S | 17 | 13 | 0 | 0.21 |
| KL | 30 | R | 3 | 27 | 0 | 0.45 |
|  | 30 | S | 20 | 10 | 0 | 0.16 |
| KS | 30 | R | 6 | 17 | 7 | 0.52 |
|  | 30 | S | 6 | 24 | 0 | 0.40 |
| P | 30 | R | 10 | 8 | 12 | 0.70 |
|  | 30 | S | 21 | 9 | 0 | 0.15 |
| K | 30 | S | 30 | 0 | 0 | 0.00 |
| SB | 30 | S | 13 | 17 | 0 | 0.28 |
| S | 30 | R | 4 | 26 | 0 | 0.43 |
|  | 30 | S | 22 | 8 | 0 | 0.13 |

**Additional file 1: Table S5.** Frequency of the S989P mutation in the *Ae. aegypti* voltage-gated sodium channel gene within resistance and susceptible mosquitoes from nine different districts of Selangor determined using AS-PCR.

| Strain | n | Status | SS  (Homozygous) | SP (Heterozygous) | PP*  (Homozygous) | Frequency of P alleles |
| --- | --- | --- | --- | --- | --- | --- |
| Bora | 30 | S | 30 | 0 | 0 | 0.00 |
| Hulu Selangor | 30 | R | 6 | 17 | 7 | 0.52 |
|  | 30 | S | 7 | 23 | 0 | 0.38 |
| Gombak | 30 | R | 3 | 22 | 5 | 0.53 |
|  | 30 | S | 3 | 27 | 0 | 0.45 |
| Hulu Langat | 30 | R | 6 | 24 | 0 | 0.40 |
|  | 30 | S | 4 | 26 | 0 | 0.43 |
| Kuala Langat | 30 | R | 0 | 30 | 0 | 0.50 |
|  | 30 | S | 0 | 30 | 0 | 0.50 |
| Kuala Selangor | 30 | R | 2 | 21 | 7 | 0.58 |
|  | 30 | S | 0 | 30 | 0 | 0.50 |
| Petaling | 30 | R | 3 | 20 | 7 | 0.56 |
|  | 30 | S | 2 | 28 | 0 | 0.46 |
| Klang | 30 | S | 3 | 27 | 0 | 0.45 |
| Sabak Bernam | 30 | S | 1 | 29 | 0 | 0.48 |
| Sepang | 30 | R | 0 | 30 | 0 | 0.50 |
|  | 30 | S | 3 | 27 | 0 | 0.45 |

.
