# Supplementary material for: Enzymatic and molecular characterization of insecticide resistance mechanisms in field populations of Aedes aegypti from Selangor, Malaysia
Source: Parasit Vectors. 2019 May 16;12:236. doi: 10.1186/s13071-019-3472-1 (PMC6521414; doi:10.1186/s13071-019-3472-1)

**Additional file 2**

**Additional file 2: Figure S1.** Gel electrophoresis of AS-PCR products corresponding to the *Ae. aegypti* sodium channel gene mutation. **a** F1534C mutation: each of the three genotypes is shown. Lane 1: ultra-low range DNA ladder; Lane 2: wild-type homozygous (FF); Lane 3: heterozygous (FC); Lane 4: mutant homozygous (CC); Lane 5: negative control. **b** V1016G mutation: Lane 1: ultra-low range DNA ladder marker; Lane 2: mutant homozygous (GG); Lane 3: heterozygous (VG); Lane 4: wild-type homozygous (VV); Lane 5: negative control. **c** S989P mutation: Lane 1: 100 bp DNA ladder marker; Lanes 2, 3: wild-type homozygous (SS); Lanes 4, 5: heterozygous (SP); Lanes 6, 7: mutant homozygous (PP); Lanes 8, 9: negative control.


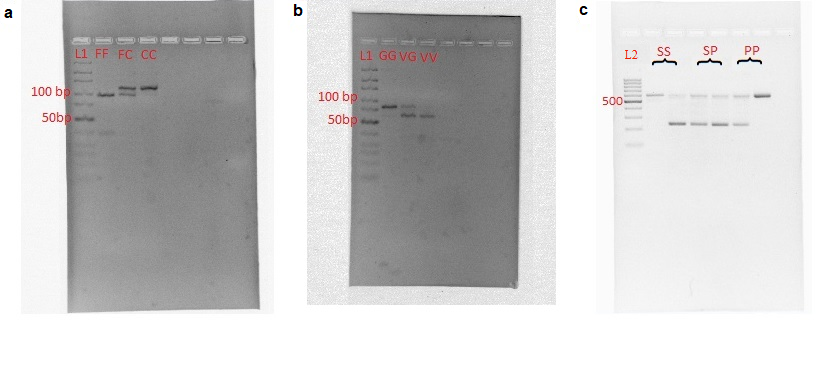


**Additional file 2: Figure S2.** Genotype sequence of V1016G mutation.


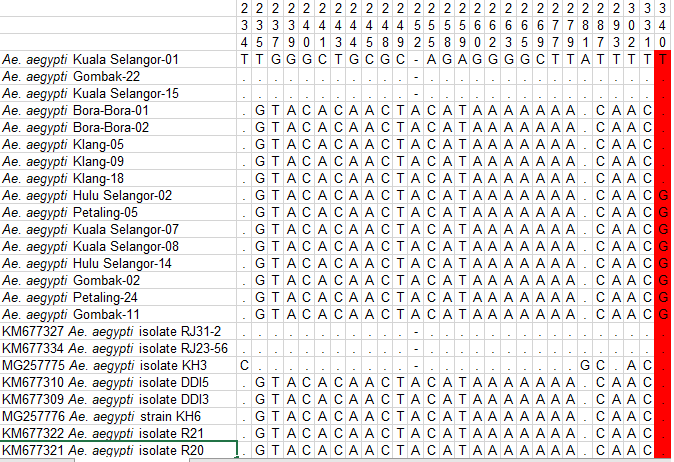


**Additional file 2: Figure S3.** Genotype sequence of S989P mutation.


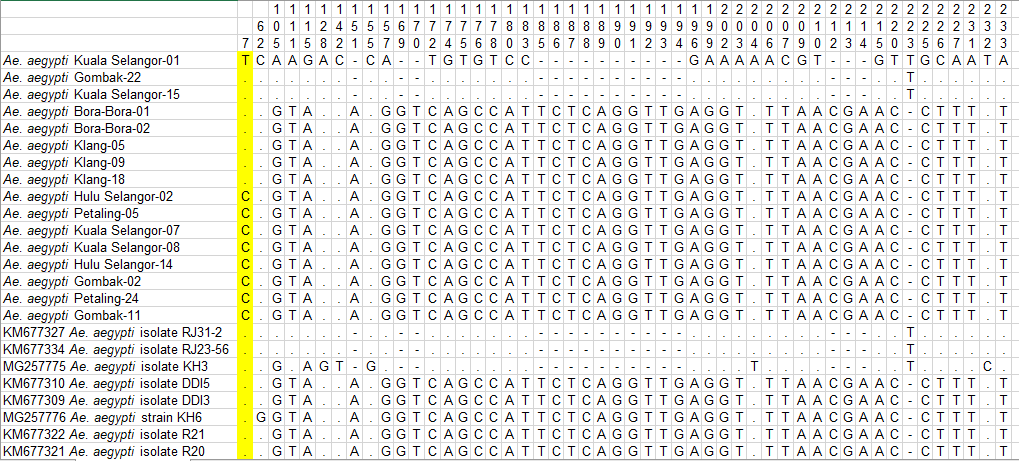


**Additional file 2: Figure S4.** Genotype sequence of F1534C mutation.


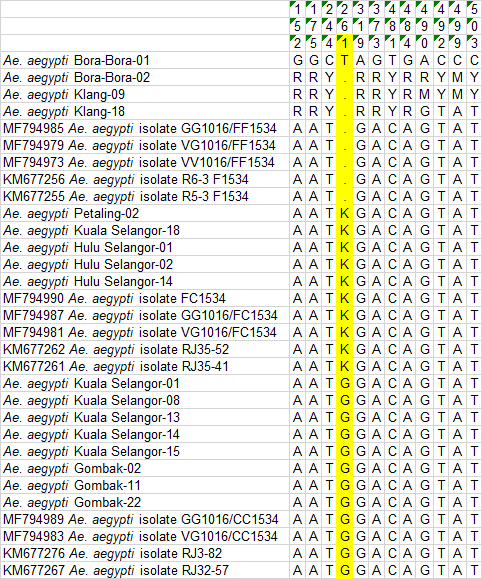

Supplement: Supplementary file 2 — Additional file 2: Figure S1. Gel electrophoresis of AS-PCR products corresponding to the Ae. aegypti sodium channel gene mutation. a F1534C mutation: each of the three genotypes is shown. Lane 1: ultra-low range DNA ladder; Lane 2: wild-type homozygous (FF); Lane 3: heterozygous (FC); Lane 4: mutant homozygous (CC); Lane 5: negative control. b V1016G mutation: Lane 1: ultra-low range DNA ladder marker; Lane 2: mutant homozygous (GG); Lane 3: heterozygous (VG); Lane 4: wild-type homozygous (VV); Lane 5: negative control. c S989P mutation: Lane 1: 100 bp DNA ladder marker; Lanes 2, 3: wild-type homozygous (SS); Lanes 4, 5: heterozygous (SP); Lanes 6, 7: mutant homozygous (PP); Lanes 8, 9: negative control. Figure S2. Genotype sequence of V1016G mutation. Figure S3. Genotype sequence of S989P mutation. Figure S4. Genotype sequence of F1534C mutation. [file 13071_2019_3472_MOESM2_ESM.docx]
